# Supplementary material for: Cost-effectiveness analysis of adding transarterial chemoembolisation to lenvatinib as first-line treatment for advanced hepatocellular carcinoma in China
Source: BMJ Open. 2023 Sep 26;13(9):e074245. doi: 10.1136/bmjopen-2023-074245 (PMC10533713; doi:10.1136/bmjopen-2023-074245)
Supplement: Supplementary data [file bmjopen-2023-074245supp001.pdf]

Supplementary Table S1: Fit parameters, AIC and BIC values for each model

			LEN-TACE				LEN			
**Index**	**Distribution**	**Parameter**	**Est**	**Se**	**AIC**	**BIC**	**Est**	**Se**	**AIC**	**BIC**
**PFS**	Log-logistic	shape	3.2270	0.2480	758.94*	765.21*	2.9320	0.2030	815.61*	821.86*
		scale	10.8880	0.4870			6.6220	0.3100		
	Gompertz	shape	0.1143	0.0152	806.42	812.70	0.0772	0.0145	879.20	885.45
		rate	0.0271	0.0048			0.0762	0.0099		
	Weibull	shape	2.1600	0.1540	775.03	781.30	1.7390	0.1050	841.79	848.04
		scale	13.7820	0.5960			8.8380	0.4360		
	Log-normal	meanlog	2.3910	0.0470	762.04	768.31	1.8878	0.0486	817.74	823.99
		sdlog	0.5513	0.0371			0.6067	0.0360		
	Gamma	shape	3.9865	0.4942	764.58	770.86	3.0411	0.3359	826.29	832.54
		rate	0.3246	0.0457			0.3888	0.0481		
	Exponential	rate	0.0671	0.0063	853.26	856.40	0.1198	0.0100	901.13	904.25
	Gen-gamma	mu	2.4161	0.0739	763.85	773.26	1.8818	0.0776	819.73	829.10
		sigma	0.5424	0.0421			0.6073	0.0365		
		Q	0.1055	0.2432			-0.0207	0.2082		
	Royston/Parmar spline model(scale=normal, 0 kont)	gamma0	-4.3370	0.2890	762.04	768.31	-3.1117	0.1961	817.74	823.99
		gamma1	1.8140	0.1220			1.6483	0.0978		
	Royston/Parmar spline model(scale=normal, 1 kont)	gamma0	-4.0295	0.5378	763.60	773.00	-3.0669	0.3074	819.71	829.08
		gamma1	1.6159	0.3179			1.6082	0.2342		
		gamma2	-0.0896	0.1345			-0.0128	0.0682		
	Royston/Parmar spline model(scale=normal, 2 kont)	gamma0	-3.4563	0.5592	762.03	774.57	-2.7900	0.3280	818.99	831.49
		gamma1	1.1548	0.3642			1.2590	0.2970		
		gamma2	-1.7142	0.8676			-0.7910	0.4710		
		gamma3	2.0204	1.0765			0.8290	0.5030		
	Royston/Parmar spline model(scale=normal, 3 kont)	gamma0	-3.3430	0.5660	763.68	779.36	-2.8390	0.3470	820.77	836.39
		gamma1	1.0410	0.3910			1.3370	0.3380		
		gamma2	-1.9230	1.9900			0.1340	1.4000		
		gamma3	1.7270	4.0660			-1.3300	2.5880		
		gamma4	0.2800	2.7960			1.3640	1.4860		
	Royston/Parmar spline model(scale=normal, 4 kont)	gamma0	-3.3579	0.5768	765.68	765.68	-2.9480	0.3750	821.45	840.20
		gamma1	1.0583	0.4067			1.5200	0.3900		
		gamma2	-1.3814	3.7579			1.9930	1.8950		
		gamma3	-0.3330	11.2152			-6.3750	5.798		
		gamma4	1.7765	11.3547			4.8450	5.7250		
		gamma5	0.0639	4.6610			-0.2470	1.9700		
	Royston/Parmar spline model(scale=normal, 5 kont)	gamma0	-3.3840	0.5890	766.75	766.75	-3.1760	0.4280	820.02	841.89
		gamma1	1.0830	0.4220			1.8950	0.4830		
		gamma2	1.3230	5.3950			4.0920	1.9290		
		gamma3	-12.2830	19.0650			-12.5980	6.4900		
		gamma4	17.4970	22.9360			15.0020	10.1850		
		gamma5	-10.8000	15.3220			-8.4620	7.7910		
		gamma6	5.0130	7.5430			2.6350	2.7250		
**OS**	Log-logistic	shape	3.3020	0.3160	582.22*	588.49*	2.9400	0.2380	723.78*	730.03*
		scale	17.9700	0.9050			11.7940	0.5960		
	Gompertz	shape	0.1391	0.0180	598.10	604.37	0.0811	0.0145	773.36	779.61
		rate	0.0073	0.0020			0.0298	0.0052		
	Weibull	shape	2.6100	0.2420	584.79	591.06	1.9440	0.1450	743.98	750.22
		scale	21.1940	0.9790			15.4080	0.7770		

Supplementary Table S1: Fit parameters, AIC and BIC values for each model

			LEN-TACE				LEN			
**Index**	**Distribution**	**Parameter**	**Est**	**Se**	**AIC**	**BIC**	**Est**	**Se**	**AIC**	**BIC**
	Log-normal	meanlog	2.8937	0.0569	585.65	591.92	2.4786	0.0538	726.80	733.05
		sdlog	0.5573	0.0470			0.6080	0.0431		
	Gamma	shape	4.4499	0.6680	583.19	589.47	3.3508	0.4286	732.93	739.18
		rate	0.2273	0.0408			0.2445	0.0367		
	Exponential	rate	0.0331	0.0038	654.31	657.45	0.0589	0.0058	799.13	802.25
	Gen-gamma	mu	2.9861	0.0734	585.14	594.55	2.4493	0.0893	728.63	738.00
		sigma	0.4595	0.0726			0.6167	0.0481		
		Q	0.5504	0.3377			-0.1120	0.2702		
	Royston/Parmar spline model(scale=normal, 0 kont)	gamma0	-5.1930	0.4100	585.65	591.92	-4.0770	0.2830	726.80	733.05
		gamma1	1.7950	0.1510			1.6450	0.1170		
	Royston/Parmar spline model(scale=normal, 1 kont)	gamma0	-3.9839	0.6927	583.99	593.40	-4.0452	0.5118	728.79	738.16
		gamma1	1.1578	0.3387			1.6253	0.2871		
		gamma2	-0.4984	0.2530			-0.0106	0.1433		
	Royston/Parmar spline model(scale=normal, 2 kont)	gamma0	-3.9100	0.7600	586.01	598.55	-3.3200	0.5010	723.12	735.61
		gamma1	1.1080	0.4000			1.0510	0.3100		
		gamma2	-0.5850	1.6860			-2.4310	0.8620		
		gamma3	0.2710	2.6460			3.0190	1.0760		
	Royston/Parmar spline model(scale=normal, 3 kont)	gamma0	-4.4770	0.9470	585.36	601.04	-3.5370	0.5570	721.47	737.09
		gamma1	1.4830	0.5240			1.2520	0.3600		
		gamma2	3.2720	2.2140			2.3380	2.5060		
		gamma3	-13.3810	8.0390			-8.6030	5.0560		
		gamma4	13.1590	8.3970			8.1020	3.3600		
	Royston/Parmar spline model(scale=normal, 4 kont)	gamma0	-4.4870	0.9800	587.42	587.42	-3.7100	0.6260	721.69	740.43
		gamma1	1.4910	0.5490			1.4040	0.4300		
		gamma2	2.6710	2.5600			1.6380	2.4290		
		gamma3	-5.4500	15.3950			-2.2890	8.3800		
		gamma4	-4.4210	25.1920			-6.9260	11.6520		
		gamma5	11.0260	16.5950			10.0550	6.3980		
	Royston/Parmar spline model(scale=normal, 5 kont)	gamma0	-4.3490	0.9860	589.24	589.24	-3.9170	0.6910	719.66	741.52
		gamma1	1.3990	0.5610			1.5940	0.4900		
		gamma2	0.2810	2.9780			4.2290	2.8250		
		gamma3	9.2650	16.6470			-15.6820	11.1400		
		gamma4	-23.8210	38.8820			25.5560	19.8710		
		gamma5	9.1510	45.3570			-32.2010	20.6110		
		gamma6	10.8110	27.9880			21.7010	10.4480		

*, best fitted model; PFS, progression-free survival; OS, overall survival; AIC, Akaike information criterion; BIC, Bayesian information criterion.

Supplementary Table S2: Validation of modeled PFS and OS data using internal and external data.

Curve	6-month (%)	12-month (%)	24-month (%)	36-month (%)
**PFS**				
LEN-TACE				
log-logistic	87.2	42.2	7.2	2.1
weibull	84.7	47.6	3.6	0.035
royston/parmar spline model (knot=5)	88.5	41.7	9.5	3.2
LAUNCH	88.2	39.2	11.4	NA
LEN				
log-logistic	57.2	14.9	2.2	0.7
weibull	60.0	18.2	0.3	0.001
royston/parmar spline model (knot=5)	56.9	14.2	2.9	1.0
LAUNCH	54.8	14.3	2.5	NA
**OS**				
LEN-TACE				
log-logistic	97.4	79.1	27.8	9.2
weibull	96.4	79.7	25.1	1.9
royston/parmar spline model (knot=5)	96.7	80.9	28.3	12.1
Chen Song et al, 2022	84.9	63.5	18.5	9.5
LAUNCH	95.9	81.5	26.1	NA
LEN				
log-logistic	87.9	48.7	11.0	3.6
weibull	85.2	54.1	9.4	0.6
royston/parmar spline model (knot=2)	89.8	45.0	16.2	8.0
REFLECT	82.1	55.0	29.8	13.0
LAUNCH	87.7	47.0	17.8	NA

PFS, progression-free survival; OS, overall survival; NA, not applicable.

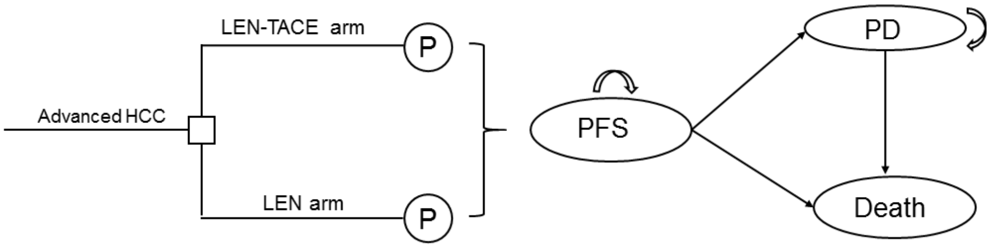

Supplementary Figure S1: Model structure of a decision tree combining the partitioned survival model. (HCC, hepatocellular carcinoma; LEN, lenvatinib; TACE, transarterial chemoembolization; PFS, progression-free survival; PD, progressed survival).

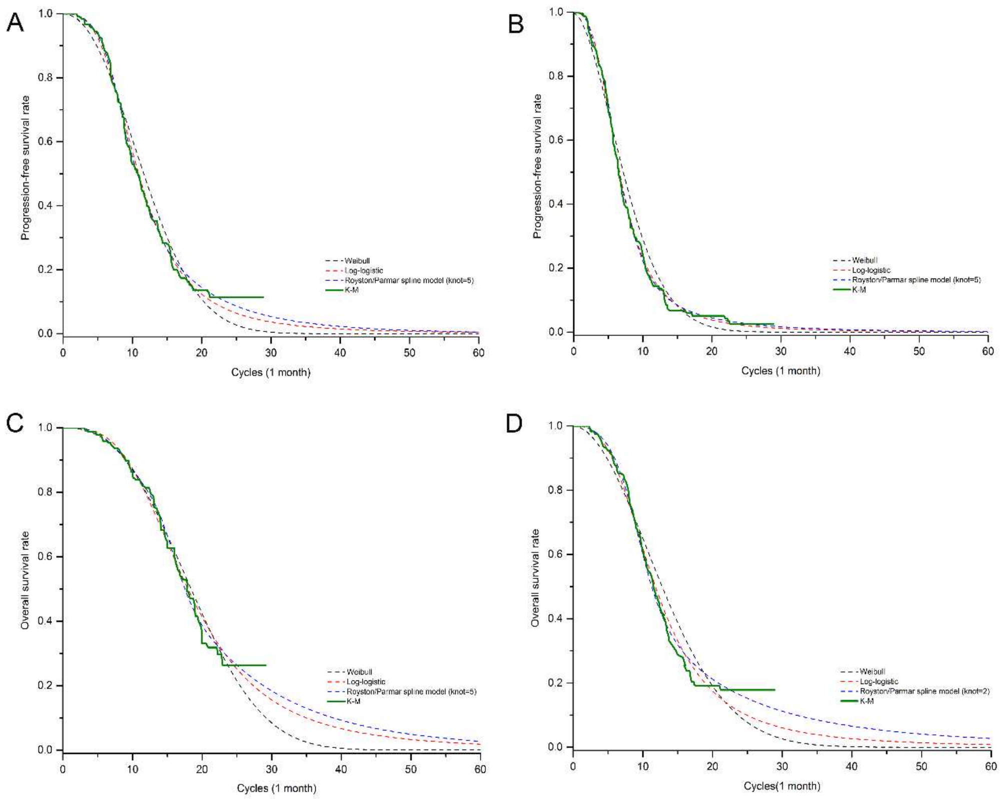

Supplementary Figure S2: The exploration and fitting of PFS and OS curves in the partitioned survival model. (A) PFS of LEN-TACE arm, (B) PFS of LEN arm, (C) OS of LEN-TACE arm, (D) OS of LEN arm.

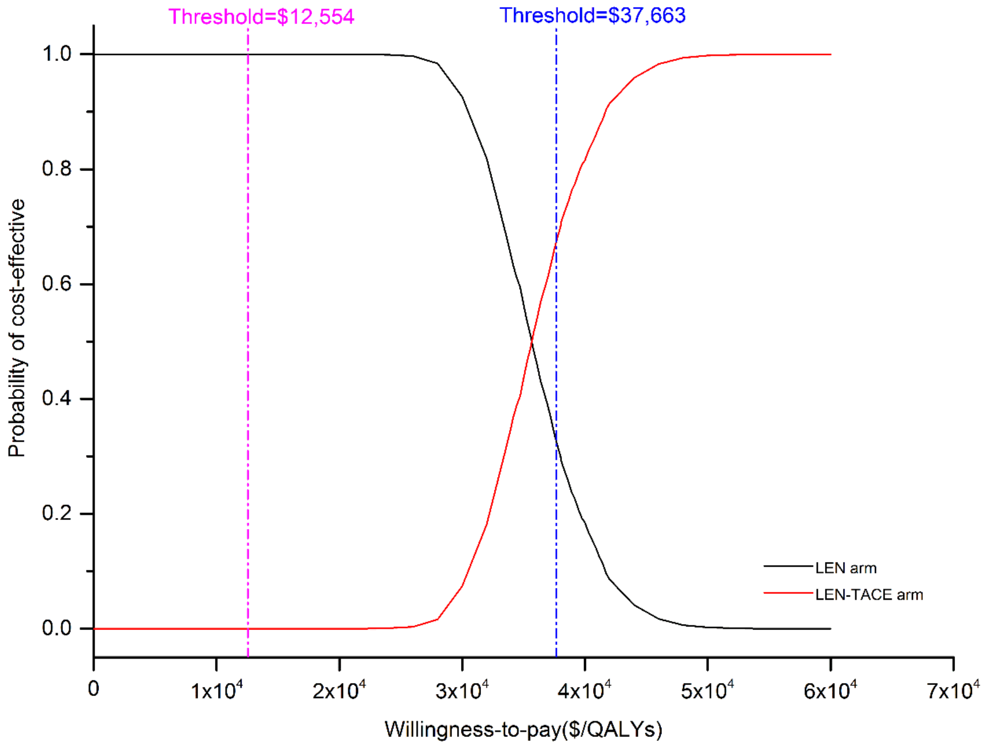

Supplementary Figure S3: Cost-effectiveness acceptability curve of LEN-TACE vs. LEN adopting Weibull distribution model. QALYs, quality-adjusted life-year.

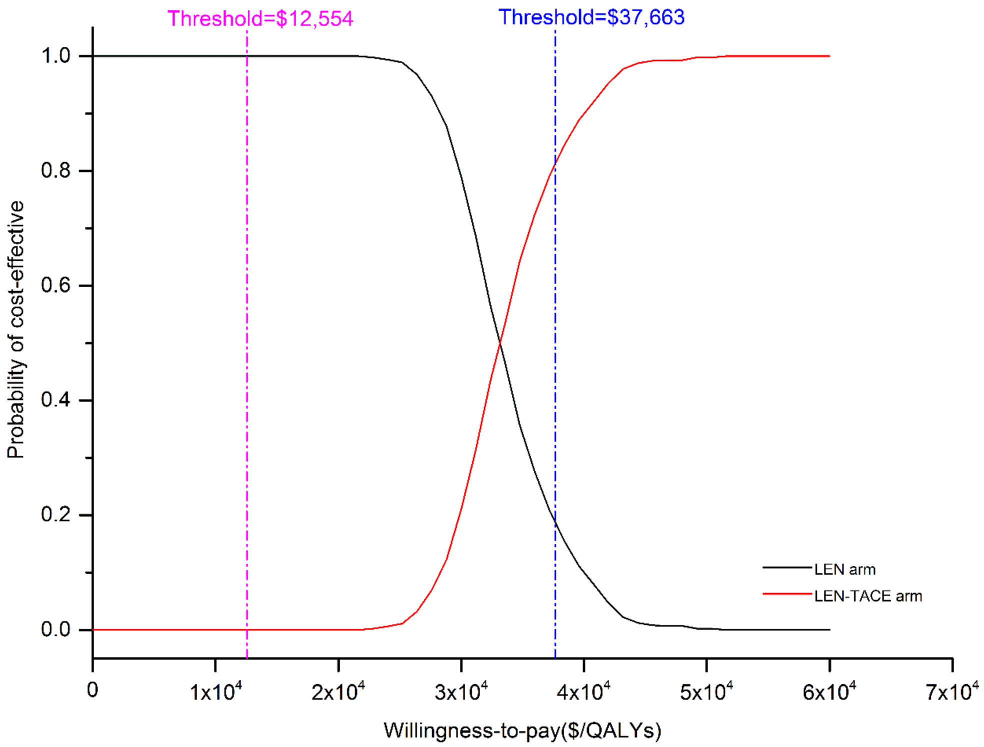

Supplementary Figure S4: Cost-effectiveness acceptability curve of LEN-TACE vs. LEN adopting Royston/Parmar spline model. QALYs, quality-adjusted life-year.
