# Supplementary material for: Cost-effectiveness analysis of adding transarterial chemoembolisation to lenvatinib as first-line treatment for advanced hepatocellular carcinoma in China
Source: BMJ Open. 2023 Sep 26;13(9):e074245. doi: 10.1136/bmjopen-2023-074245 (PMC10533713; doi:10.1136/bmjopen-2023-074245)
Supplement: Author's manuscript [file bmjopen-2023-074245.draft_revisions.pdf]

BMJ Open is committed to open peer review. As part of this commitment we make the peer review history of every article we publish publicly available.

When an article is published we post the peer reviewers' comments and the authors' responses online. We also post the versions of the paper that were used during peer review. These are the versions that the peer review comments apply to.

The versions of the paper that follow are the versions that were submitted during the peer review process. They are not the versions of record or the final published versions. They should not be cited or distributed as the published version of this manuscript.

BMJ Open is an open access journal and the full, final, typeset and author-corrected version of record of the manuscript is available on our site with no access controls, subscription charges or pay-per-view fees (http://bmjopen.bmj.com).

If you have any questions on BMJ Open's open peer review process please email info.bmjopen@bmj.com

BMJ Open

Cost-effectiveness analysis of adding transarterial chemoembolization to lenvatinib as first-line treatment for advanced hepatocellular carcinoma in China

Journal:	*BMJ Open*
Manuscript ID	bmjopen-2023-074245
Article Type:	Original research
Date Submitted by the Author:	03-Apr-2023

Complete List of Authors:	Li, Wei; Huazhong University of Science and Technology, Department of Pharmacy<br>Wan, Li; Huazhong University of Science and Technology, Department of Pharmacy

Keywords:	HEALTH ECONOMICS, Health economics < HEALTH SERVICES ADMINISTRATION & MANAGEMENT, Hepatology < INTERNAL MEDICINE, Clinical Decision-Making, Decision Making

SCHOLARONE™
Manuscripts

I, the Submitting Author has the right to grant and does grant on behalf of all authors of the Work (as defined in the below author licence), an exclusive licence and/or a non-exclusive licence for contributions from authors who are: i) UK Crown employees; ii) where BMJ has agreed a CC-BY licence shall apply, and/or iii) in accordance with the terms applicable for US Federal Government officers or employees acting as part of their official duties; on a worldwide, perpetual, irrevocable, royalty-free basis to BMJ Publishing Group Ltd ("BMJ") its licensees and where the relevant Journal is co-owned by BMJ to the co-owners of the Journal, to publish the Work in this journal and any other BMJ products and to exploit all rights, as set out in our licence.

The Submitting Author accepts and understands that any supply made under these terms is made by BMJ to the Submitting Author unless you are acting as an employee on behalf of your employer or a postgraduate student of an affiliated institution which is paying any applicable article publishing charge ("APC") for Open Access articles. Where the Submitting Author wishes to make the Work available on an Open Access basis (and intends to pay the relevant APC), the terms of reuse of such Open Access shall be governed by a Creative Commons licence – details of these licences and which Creative Commons licence will apply to this Work are set out in our licence referred to above.

Other than as permitted in any relevant BMJ Author's Self Archiving Policies, I confirm this Work has not been accepted for publication elsewhere, is not being considered for publication elsewhere and does not duplicate material already published. I confirm all authors consent to publication of this Work and authorise the granting of this licence.

For peer review only

Cost-effectiveness analysis of adding transarterial chemoembolization to lenvatinib as first-line treatment for advanced hepatocellular carcinoma in China

Wei Li, and Li Wan

Department of Pharmacy, Maternal and Child Health Hospital of Hubei Province, Tongji Medical College, Huazhong University of Science and Technology, Wuhan 430070, China

Correspondence to:

Wei Li

Department of Pharmacy, Maternal and Child Health Hospital of Hubei Province, Tongji Medical College, Huazhong University of Science and Technology, Wuhan 430070, China

liwei1988hust@126.com

For peer review only

For peer review only - http://bmjopen.bmj.com/site/about/guidelines.xhtml

Abstract

Objective The objective of this study was to evaluate the comparative cost-effectiveness of lenvatinib (LEN) plus transarterial chemoembolization (TACE) [LEN-TACE] and LEN alone to treat advanced hepatocellular carcinoma (HCC) from the perspective of the Chinese healthcare system.
Design A three-state partitioned survival model using clinical survival data from a phase III LAUNCH trial, a 5-year time horizon for costs and quality-adjusted life-years (QALYs) was constructed to analyse the cost-effectiveness of LEN-TACE. Clinical inputs were extracted from the LAUNCH trial, with outcomes extrapolated using standard and flexible parametric survival models. Costs and utilities derived from published literature were discounted at an annual rate of 5%. Sensitivity analyses, and scenario analyses were conducted to test the robustness of the model.
Setting The Chinese healthcare system perspective.
Participants A hypothetical Chinese cohort of patients with advanced HCC.
Interventions Transarterial chemoembolization plus lenvatinib versus lenvatinib.
Primary outcome measure Costs, QALYs, incremental cost-effectiveness ratio (ICER).
Results: Base-case analysis revealed that LEN-TACE would be cost-effective in China at the willingness-to-pay (WTP) threshold of $37,663 per QALYs, with improved effectiveness of 0.382 QALYs and additional cost of $12,151 (ICER: $31,808 per QALY). The probabilistic sensitivity analysis suggested that LEN-TACE had a 93.5% probability of cost-effectiveness at WTP threshold of 3 times GDP per capital ($37,663). One-way deterministic sensitivity analysis indicated that the duration of lenvatinib treatment in both two arms, utility of PFS, and the cost of TACE had a greater impact on the stability of ICER values. Scenario analyses results were in line with base-case analysis.
Conclusions: LEN-TACE might be a cost-effective strategy compared with LEN for the first-line treatment of patients with advanced HCC in China.

Strengths and limitations of this study

- The partitioned survival model acquires the proportion of patients in different health states directly from the survival curves, without too many assumptions and is commonly applied in economic evaluations of advanced or metastatic cancer.
- Both standard and flexible parametric survival models are used to predict the long term survival outcomes in this cost-effectiveness analysis.
- External data accessed through reconstructing the survival curves of previously clinical trials on HCC is used to verify our model choice.
- In this model, the proportions of patients receiving subsequent anticancer treatment originated from clinical trial data, which might not true represent the prevalence of subsequent anticancer strategy used in real-world practice.
- We do not conduct the subgroup analysis due to a short of subgroup survival data.

Keywords: cost-effectiveness, transarterial chemoembolization, lenvatinib, hepatocellular carcinoma

For peer review only

Introduction

Hepatocellular carcinoma (HCC) is the six-most commonly diagnosed cancer and ranks third in cancer-related mortality worldwide[1]. However, this situation is even worse in China, where it is the fifth most commonly diagnosed cancer and the secondary leading cause of cancer-related death [2]. It is estimated that more than 70% of HCC patients have advanced cancer at the time of diagnosis, which limits the feasibility of radical surgical treatment in advanced cases [3, 4]. Therefore, targeted systemic therapies have become a ray of hope for advanced HCC patients. Lenvatinib (LEN), an oral multiple receptor tyrosine kinase inhibitor, noninferior to sorafenib, is currently recommended as first-line treatments for advanced HCC [5, 6]. Although the median overall survival (OS) for Chinese subgroup population was longer than that of the intention-to-treat (ITT) population (15.0 vs. 13.6 months), the efficacy of LEN is still unsatisfactory [7]. To further prolong the overall survival, much endeavors have been made, such as the combination of LEN with other treatments.

Transarterial chemoembolization (TACE) can effectively reduce tumor burden by targeting intrahepatic tumors, thus may enhance the outcomes in patients with advanced HCC [8]. While, LEN is more likely to be effective in patients with lower tumor burden [9]. In terms of mechanism of action, the combination of LEN and TACE (LEN-TACE) has a synergistic effect. Recently, a series of observational studies indicate that the combination of LEN and TACE brings favorable survival outcomes [10-12]. However, these observation studies only provide limited supporting evidence and need to be further verified by large-scale clinical trial.

Nowadays, a Chinese-patient-based phase III trial, LAUNCH (NCT03905967), demonstrates that LEN-TACE prolongs median OS by a significant 6.3 months, compared with LEN monotherapy in the ITT population [13]. Additionally, LEN-TACE shows a statistically significant improvement in progression-free survival (PFS) time, with a median improvement in PFS of 4.2 months [13]. Although the remarkable results of this trial raises hope for patients extend survival, higher medical costs generated by combination therapy may impose a heavy socioeconomic burden on patients and the healthcare system, especially in resource-limiting settings like China. At present, economic evaluations of LEN in the area of HCC mainly focus on the comparision of intervention regimes, such as the LEN vs. LEN similar drugs (sorafenib, donafenib) [14, 15], LEN vs. sintilimab plus bevacizumab [16]. There is no cost-effectiveness evaluation of LEN-TACE strategy in patients with advanced HCC from the perspective of Chinese healthcare system. We thus compare the cost-effectiveness of the two strategies to treat advanced HCC by using model data from LAUNCH. These findings provide evidence for use by HCC patients and the physicians treating them, as well as health policymakers.

Methods

Model Structure and Outcomes

A partitioned survival model (PSM), including three mutually exclusive disease-related health states: progression-free (PFS), progressed disease (PD), and death, was constructed in TreeAge Pro 2020 (TreeAge Software, Williamstown, MA) to simulate the cohort of patients in LAUNCH trial [13]. The model cycle length was 1 month, and the time horizon was 5 years.

For peer review only - http://bmjopen.bmj.com/site/about/guidelines.xhtml

Our target population was patient with primary advanced HCC receiving first-line treatments in China. These patients underwent lenvatinib (LEN) alone or plus TACE (LEN-TACE) during the simulated time. The tree diagram and bubble diagram were illustrated in **Supplementary Figure S1**. It was hypothesized that the patient cohort entered the model in the PFS state and then either occupied in the same state or moved to the other states according to transition probabilities during each model cycle. The initial age of the simulated cohort population was set to 55 years old, which was consistent with the average age of the LAUNCH trial [13].

The primary model outcomes were the corresponding total costs of two therapeutic regimens, quality-adjusted life years (QALYs), and the incremental cost-effectiveness ratios (ICERs). Both costs and utility values were discounted at 5% annually for base-case analysis, according to guideline for health economic evaluations in China [17]. All costs were converted into 2021 US dollars (US 1$ = CNY ¥6.45). Three-fold of the per capita gross domestic product (GDP) of China in 2021 (US$37,663/QALYs) was used as the willingness-to-pay (WTP) threshold to evaluate the cost-effectiveness of the two competing strategies [17].

Clinical Data and Model Probabilities

The clinical efficacy and safety data of LAUNCH trial [13] were used to explore the cost-effectiveness of lenvatinib with or without TACE for advanced HCC. The PFS and OS data were extracted from the published K-M curves by using GetData Graph Digitizer software (version 2.26) and then was utilized to reconstruct the individual patient data (IPD) according to Guyot's method [18]. To extrapolate survival outcomes outside the observation period, different parameter distributions (exponential, weibull, gompertz, log-logistic, log-normal, gamma, gen-gamma and royston/parmar spline model) were employed to fit the reconstructed IPD. The choice of survival model to use was based on statistical goodness of fit (using the Akaike's information criterion (AIC) and the Bayesian information criterion (BIC)), visual inspection and clinical plausibility of the extrapolations. Finally, the log-logistics model had the best AIC and BIC was used for both arms for PFS and OS in base-case analysis (**see Supplementary Table S1**). We also provided the exploration and fitting of PFS and OS curves for visual inspection (**see Supplementary Figure S2**). The impact of selecting alternative survival model was investigated in scenario analyses.

Cost and Utility

Only direct medical costs, including the first-line and subsequent treatment cost, monitoring cost, hepatectomy cost, TACE cost, management of serious adverse events (SAEs, grade 3 to 4), follow-up cost, and cost of terminal care in end of life, were calculated from the perspective of the Chinese healthcare system. It should be noted that, follow-up costs included CT examination, urinalysis, blood test, and blood biochemical examination; monitoring costs included diagnosis fee, nursing fee, injection fee, and bed fee, more details were presented in **Table 1**. Based on the LAUNCH trial [13], the median number of TACE sessions per patient was 3 (range, 1-6 sessions), once every 8 weeks. All costs were derived from previously published literature [19-22]. To calculate the costs in base-case analysis, we made the following assumptions in this model:

a) We assumed that the average weight of a patient was 60 Kg.
b) We did not consider any cause of dose reduction or interruption of lenvatinib in base-case analysis. Patients were assumed to receive full dose lenvatinib (body weight ≥ 60

For peer review only - http://bmjopen.bmj.com/site/about/guidelines.xhtml

Kg, 12mg daily; body weight < 60 Kg, 8mg daily) until disease progression or unacceptable toxicity.

c) In line with the LAUNCH trial protocol, patients after progression or discontinuation would receive subsequent treatments, such as, curative surgical resection, tyrosine kinase inhibitor (TKI) therapy, programmed cell death protein-1 (PD-1) inhibitor and so on. The selection of specific TKI and PD-1inhibitor as subsequent treatment was based on the guideline of China [23]. For simplification, we assumed that patients in LEN-TACE arm would receive one of the following subsequent treatments: hepatectomy, regorafenib (160 mg/day, 3 weeks of medications, then discontinuing for 1 week), tislelizumab (200mg/day, q3w), best supportive care (BSC); Patients in LEN arm would receive one of the following subsequent treatments: regorafenib (160 mg/day, 3 weeks of medications, then discontinuing for 1 week), tislelizumab (200mg/day, q3w), TACE, BSC. The proportion of patients receiving subsequent therapy was derived from the LAUNCH trial.

d) To better reflect the cost of real-world clinical practice, the duration of treatment in PFS state was considered. The cost for lenvatinib was charged based on the upper limit of the treatment duration, as per the LAUNCH trial. In addition, a 2-year maximum treatment duration of tislelizumab was taken into consideration based on previous study [24].

e) Only grade 3 or 4 SAEs with an incidence of >5% were considered, including elevated alanine aminotransferase (ALT)/ aspartate aminotransferase (AST), hypertension, decreased weight, diarrhea, and hyperbilirubinemia. The 3-4 SAEs-related costs were calculated once in the first cycle by multiplying the incidence of the SAEs by the costs of managing the SAEs per event.

f) Given that the subsequent treatment information was limited from the LAUNCH trial records. The grade 3-4 SAEs-related costs for subsequent treatment were ignored.

g) We assumed that all the patients received terminal care three months before they died in the base-case analysis.

Health state utility values were derived from previously published cost-effectiveness studies regarding Chinese patients with unresectable HCC, and the values were set at 0.76 for the PFS, 0.68 for PD state respectively [25]. Considering that the occurrence of adverse events might have impact on patients' health-related quality of life. This present study also considered the disutility due to adverse events, including elevated ALT/AST, hypertension, decreased weight, diarrhea, and hyperbilirubinemia, which were obtained from previous researches [15, 19]. The details were listed in **Table 2**.

Sensitivity Analysis and Scenario Analysis

One-way deterministic (DSA) and probabilistic sensitivity analysis (PSA) were performed to examine the robustness of model outcomes. In one-way sensitivity analysis, relevant parameters that had substantial impact on ICER, were tested orderly at the upper and lower limits of plausible ranges, which were listed and illustrated in **Table 1**. It should be noted that a bodyweight less than 60 Kg associated with lower lenvatinib dose (8 mg daily), resulting in the difference in treatment costs should not be ignored. Hence, we assumed a HCC patient weighted less than 60 Kg (lenvatinib, 8 mg daily) to investigate the impact on model outcomes. The results of DSA were graphed in the tornado diagram. PSA was performed to determine the effects of uncertainty in all model parameters simultaneously via 1000 Monte Carlo simulations, which were illustrated in scatter plot and cost-effectiveness acceptability curves (CEACs). The key parameters input our model were shown in **Table 1 and Supplementary Table S1**.

For peer review only - http://bmjopen.bmj.com/site/about/guidelines.xhtml

We also conducted two scenario analyses to explore the impact of different key model setting and assumptions on the economic outcomes. To eradicate the uncertainty caused by the extrapolation of the survival curves, we used different parametric distribution survival models (i.e. weibull and royston/parmar spline model) to estimate the ICER. The weibull distribution obtained lower survival benefit compared to that of other distributions in this study, so it was suitable to examine the model stability under extreme conditions. The parameter values of weibull distribution and royston/parmar spline model for PFS and OS curves were exhibited in **Supplementary Table S1**.

Patient and public involvement

No patients or public were involved in the study.

Results

Base-Case Analysis

In our base-case analysis, LEN-TACE cost $31,394 and yielded 1.166 QALYs, whereas LEN cost $19,243 and yielded 0.784 QALYs (**see Table 2**). The ICER of LEN-TACE vs. LEN was $31,808 per QALY, which was below the WTP threshold ($37,663/QALYs), demonstrating that the LEN-TACE was a cost- effective treatment option for advanced HCC.

Sensitivity Analysis and Scenario Analysis

DSA result indicated that the model was most sensitive to the duration of lenvatinib treatment in both two arms, utility of PFS, and the cost of TACE. We only presented the top 10 sensitive factors in **Figure 1**. All parameters fluctuated within the range in the DSA did not bring the ICER values surpass the WTP threshold ($37,663/QALYs). When the duration of lenvatinib treatment in both two arms increased from 5 to 12 months in this model, the ICER went up from $20,508 to $31,808 per QALYs correspondingly. For patient with a bodyweight <60 Kg, namely the lenvatinib daily dose reduced from 12 to 8 mg, the ICER dropped from $31,808 to $27,559 per QALYs. In other words, for patients with lower bodyweight, LEN-TACE had more favorable economic outcomes in comparison with LEN. As shown in **Figure 2**, most of scatter points were between the line of 1- and 3-fold GDP. The CEACs also indicated that the probability of LEN-TACE being cost-effective was about 93.5% at the threshold of $37,663/QALYs, which was consistent with those of base-case analysis outcomes (**Figure 3**).

Assuming alternative weibull and royston/parmar spline model would lead to the slight increase of ICER values ($35,780, 33,540 vs. $31,808) compared with that of log-logistic model, respectively (**Table 2**). Overall, the results of scenario analyses were congruous with the conclusions of the base-case analysis, confirming the robustness of our model outcomes. The CEACs of scenario analyses were given in **Supplementary Figure S3-4**.

Model Validation

External data accessed through reconstructing the survival curves of previously clinical trials [26, 27] on HCC was used to verify our model choice. Validation results for modeled PFS and OS data were presented in **Supplementary Table S2 and Supplementary Figure S2**. The 6, 12, 24, 36-month survival rate estimated by best-fitting distribution and alternative models

For peer review only - http://bmjopen.bmj.com/site/about/guidelines.xhtml

compared with the results that observed in LAUNCH trial and external data were presented in **Supplementary Table S2 and Supplementary Figure S2**.

Discussion

In this study, we demonstrated that LEN-TACE might be a cost-saving treatment option for advanced HCC patients, with ICER versus LEN of $31,808 per QALYs. PSA results suggested that the probability of LEN-TACE being cost-effective was 93.5% at the WTP threshold of $37,663/QALYs, which proved the robustness of the base-case analysis results. When choosing alternative survival models, such as weibull and royston/parmar spline model, the conclusion of LEN-TACE strategy being cost-effective was unchanged with a probability of 68.4% and 83.0%, respectively (**Supplementary Figure S3-4**).

The DSA results revealed that the ICER was most sensitive to the duration of lenvatinib treatment in both two arms, utility of PFS, and the cost of TACE. However, none of parameter variation could lead to an inversion of this economic evaluation results. It also should be noted that the utility value of PFS varying within a relatively wide range (0.61-0.91) would lead to a very close to the threshold of WTP. However, if we input a narrowed PFS utility value variation range (0.73-0.76) as reported by NICE single technology appraisal [28] in our model, the obtained ICER ($31,804-32,759/QALYs) was significantly below the threshold line.

The choice of log-logistic model as survival distribution for extrapolation in base-case analysis was consistency with previous findings of extrapolation in advanced HCC [29]. The log-logistic model used for OS in LEN-TACE arm estimated a 3-year survival rate of 9.2% compared with that of 9.5%, reported by a retrospective study [26] (median follow-up time, 27 months) in China. Although the log-logistic model used for OS in LEN arm predicted a 3-year survival rate of 3.6%, which was not consistent with that of 13% reported in REFLECT trial [27], we thought that this model exhibited a reasonable fit to the observed data. The reasons were as follows: (a) There were some differences in baseline patient characteristics between LAUNCH and REFLECT trial, especially in α-fetoprotein and Barcelona Clinic Liver Cancer stage. (b) The higher α-fetoprotein level and more advanced Barcelona Clinic Liver Cancer stage in LAUNCH trial might be associated with lower OS survival rate. (c) An alternative royston/parmar spline model (knot=2) projected a higher 3-year OS survival rate of 8.0%, which was more close to the REFLECT trial, but it did not change the economic outcomes.

To our knowledge, this is the first study to assess the economic outcomes of adding TACE to lenvatinib as a first-line treatment for advanced HCC in China. The findings of this study suggested that the addition of TACE to lenvatinib as an alternative treatment option for Chinese patients with advanced HCC might shed light on a potentially cost-effective practice-changing opportunity for clinicians, researchers, and policymakers in the future.

A number of existing limitations in this study should be noted. First, due to the lack of utility based on only local populations, the EQ-5D utilities of the PFS and PD states deriving from previously published cost-effectiveness analysis considering advanced HCC were used in this model, which may bring bias. We will update our model outcomes when these data are available in China. Second, the health outcomes beyond the follow-up period were estimated by parametric distributions, and the selection of the best fit distribution largely depend on the long follow-up external data. Inappropriate choice of parameter models would result in

For peer review only - http://bmjopen.bmj.com/site/about/guidelines.xhtml

overestimation or underestimation of survival rate, which in turn, brought bias to the economic evaluation. Third, the proportions of patients receiving subsequent anticancer treatment originated from clinical trial data, which might not true represent the prevalence of subsequent anticancer strategy used in real-world practice. Fourth, we did not conduct the subgroup analysis due to a short of subgroup PFS and OS data.

Conclusions

The findings of this economic evaluation suggest that the combination of lenvatinib and TACE is likely to be a cost-effectiveness treatment option for Chinese patients with advanced HCC under the WTP threshold of $37,663 in China. In the future, further long-term follow-up data and real-world data are needed to verify the robustness of model outcomes.

Acknowledgements

None.

Contributors

WL contributed to the study concepts and design, analysis and interpretation of the data, drafting and revising of the paper; LW contributed to the review of the paper and supervision; all authors approved of final manuscript; all authors agree to be accountable for all aspects of the work.

Funding

This work was supported by the Maternal and Child Health Hospital of Hubei Province Research Project (No. 2021SFYM030).

Competing interests

The authors declare that there is no conflict of interest.

Patient consent for publication

Not required.

Ethics approval

Ethical approval was not necessary, because our economic evaluation is based on a mathematical model analysis, and does not contain any studies with human participants or animals performed.

Provenance and peer review

Not commissioned; externally peer reviewed.

For peer review only

Data availability statement

Data are available upon reasonable request.

Supplemental material

Supplemental material for this article is available online.

References

1. Sung H, Ferlay J, Siegel RL, et al. Global Cancer Statistics 2020: GLOBOCAN Estimates of Incidence and Mortality Worldwide for 36 Cancers in 185 Countries. *CA Cancer J Clin* 2021; 71: 209-249. 2021/02/05. DOI: 10.3322/caac.21660.

2. International Agency for Research on Cancer. (2020). All Cancer Source: Globocan 2020. Available online at: https://gco.iarc.fr/ (accessed September 8, 2021).

3. Llovet JM and Bruix J. Novel advancements in the management of hepatocellular carcinoma in 2008. *J Hepatol* 2008; 48 Suppl 1: S20-37. 2008/02/29. DOI: 10.1016/j.jhep.2008.01.022.

4. Park JW, Chen M, Colombo M, et al. Global patterns of hepatocellular carcinoma management from diagnosis to death: the BRIDGE Study. *Liver Int* 2015; 35: 2155-2166. 2015/03/11. DOI: 10.1111/liv.12818.

5. Benson AB, D'Angelica MI, Abbott DE, et al. Hepatobiliary Cancers, Version 2.2021, NCCN Clinical Practice Guidelines in Oncology. *J Natl Compr Canc Netw* 2021; 19: 541-565. 2021/05/25. DOI: 10.6004/jnccn.2021.0022.

6. Chen LT, Martinelli E, Cheng AL, et al. Pan-Asian adapted ESMO Clinical Practice Guidelines for the management of patients with intermediate and advanced/relapsed hepatocellular carcinoma: a TOS-ESMO initiative endorsed by CSCO, ISMPO, JSMO, KSMO, MOS and SSO. *Ann Oncol* 2020; 31: 334-351. 2020/02/19. DOI: 10.1016/j.annonc.2019.12.001.

7. Kudo M, Finn RS, Qin S, et al. Lenvatinib versus sorafenib in first-line treatment of patients with unresectable hepatocellular carcinoma: a randomised phase 3 non-inferiority trial. *Lancet* 2018; 391: 1163-1173. 2018/02/13. DOI: 10.1016/S0140-6736(18)30207-1.

8. Sieghart W, Hucke F and Peck-Radosavljevic M. Transarterial chemoembolization: modalities, indication, and patient selection. *J Hepatol* 2015; 62: 1187-1195. 2015/02/15. DOI: 10.1016/j.jhep.2015.02.010.

9. Lencioni R, de Baere T, Soulen MC, et al. Lipiodol transarterial chemoembolization for hepatocellular carcinoma: A systematic review of efficacy and safety data. *Hepatology* 2016; 64: 106-116. 2016/01/15. DOI: 10.1002/hep.28453.

10. Casadei-Gardini A, Scartozzi M, Tada T, et al. Lenvatinib versus sorafenib in first-line treatment of unresectable hepatocellular carcinoma: An inverse probability of treatment weighting analysis. *Liver Int* 2021; 41: 1389-1397. 2021/02/07. DOI: 10.1111/liv.14817.

11. Fu Z, Li X, Zhong J, et al. Lenvatinib in combination with transarterial chemoembolization for treatment of unresectable hepatocellular carcinoma (uHCC): a retrospective controlled study. *Hepatology international* 2021; 15: 663-675. 2021/04/21. DOI: 10.1007/s12072-021-10184-9.

For peer review only - http://bmjopen.bmj.com/site/about/guidelines.xhtml

12. Kawamura Y, Kobayashi M, Shindoh J, et al. Lenvatinib-Transarterial Chemoembolization Sequential Therapy as an Effective Treatment at Progression during Lenvatinib Therapy for Advanced Hepatocellular Carcinoma. *Liver cancer* 2020; 9: 756-770. 2021/01/15. DOI: 10.1159/000510299.

13. Peng Z, Fan W, Zhu B, et al. Lenvatinib Combined With Transarterial Chemoembolization as First-Line Treatment for Advanced Hepatocellular Carcinoma: A Phase III, Randomized Clinical Trial (LAUNCH). *J Clin Oncol* 2023; 41: 117-127. 2022/08/04. DOI: 10.1200/jco.22.00392.

14. Cai H, Zhang L, Li N, et al. Lenvatinib versus sorafenib for unresectable hepatocellular carcinoma: a cost-effectiveness analysis. *Journal of comparative effectiveness research* 2020; 9: 553-562. 2020/05/19. DOI: 10.2217/cer-2020-0041.

15. Meng R, Zhang X, Zhou T, et al. Cost-effectiveness analysis of donafenib versus lenvatinib for first-line treatment of unresectable or metastatic hepatocellular carcinoma. *Expert Rev Pharmacoecon Outcomes Res* 2022; 22: 1079-1086. 2022/05/18. DOI: 10.1080/14737167.2022.2079498.

16. Zhou T, Wang X, Cao Y, et al. Cost-effectiveness analysis of sintilimab plus bevacizumab biosimilar compared with lenvatinib as the first-line treatment of unresectable or metastatic hepatocellular carcinoma. *BMC health services research* 2022; 22: 1367. 2022/11/19. DOI: 10.1186/s12913-022-08661-4.

17. Chinese Pharmaceutical Association. China Guidelines for Pharmacoeconomic Evaluations. (2019). Available online at: https://www.cpa.org.cn//?do=infoandcid=75017 (accessed May 11, 2022).

18. Guyot P, Ades AE, Ouwens MJ, et al. Enhanced secondary analysis of survival data: reconstructing the data from published Kaplan-Meier survival curves. *BMC Med Res Methodol* 2012; 12: 9. 2012/02/03. DOI: 10.1186/1471-2288-12-9.

19. Li M, Lin S, Wilson L, et al. Cost-Effectiveness Analysis of Hepatic Arterial Infusion of FOLFOX Combined Sorafenib for Advanced Hepatocellular Carcinoma With Portal Vein Invasion. *Frontiers in oncology* 2021; 11: 562135. 2021/03/27. DOI: 10.3389/fonc.2021.562135.

20. Meng R, Cao Y, Zhou T, et al. The Cost Effectiveness of Donafenib Compared With Sorafenib for the First-Line Treatment of Unresectable or Metastatic Hepatocellular Carcinoma in China. *Frontiers in public health* 2022; 10: 794131. 2022/04/19. DOI: 10.3389/fpubh.2022.794131.

21. Zhang H, Zeng X, Peng Y, et al. Cost-Effectiveness Analysis of Hepatic Arterial Infusion Chemotherapy of Infusional Fluorouracil, Leucovorin, and Oxaliplatin Versus Transarterial Chemoembolization in Patients With Large Unresectable Hepatocellular Carcinoma. *Front Pharmacol* 2022; 13: 849189. 2022/05/14. DOI: 10.3389/fphar.2022.849189.

22. Zhao M, Pan X, Yin Y, et al. Cost-Effectiveness Analysis of Five Systemic Treatments for Unresectable Hepatocellular Carcinoma in China: An Economic Evaluation Based on Network Meta-Analysis. *Frontiers in public health* 2022; 10: 869960. 2022/05/03. DOI: 10.3389/fpubh.2022.869960.

23. National Health Commission of the People's Republic of China. Guidelines for the diagnosis and treatment of primary hepatic carcinoma (2022 edition). Available online at:

For peer review only - http://bmjopen.bmj.com/site/about/guidelines.xhtml

http://www.nhc.gov.cn/yzygj/s7659/202201/a01ceb75c62b486fa459e36ba0fdfdbc.shtml (accessed Feb 11, 2023).

24. Wu B, Zhang Q and Sun J. Cost-effectiveness of nivolumab plus ipilimumab as first-line therapy in advanced renal-cell carcinoma. *J Immunother Cancer* 2018; 6: 124. 2018/11/22. DOI: 10.1186/s40425-018-0440-9.

25. Hou Y and Wu B. Atezolizumab plus bevacizumab versus sorafenib as first-line treatment for unresectable hepatocellular carcinoma: a cost-effectiveness analysis. *Cancer communications (London, England)* 2020; 40: 743-745. 2020/11/08. DOI: 10.1002/cac2.12110.

26. Chen S, Wu Z, Shi F, et al. Lenvatinib plus TACE with or without pembrolizumab for the treatment of initially unresectable hepatocellular carcinoma harbouring PD-L1 expression: a retrospective study. *Journal of cancer research and clinical oncology* 2022; 148: 2115-2125. 2021/08/29. DOI: 10.1007/s00432-021-03767-4.

27. Okusaka T, Ikeda K, Kudo M, et al. Safety and efficacy of lenvatinib by starting dose based on body weight in patients with unresectable hepatocellular carcinoma in REFLECT. *Journal of gastroenterology* 2021; 56: 570-580. 2021/05/06. DOI: 10.1007/s00535-021-01785-0.

28. NICE Single Technology Appraisal. Lenvatinib for advanced, unresectable, untreated hepatocellular carcinoma: committee papers [TA551].2018. Available online at: https://www.nice.org.uk/guidance/ta551/evidence/appraisal-consultation-committee-paperspdf-6652960238 (Accessed Jun 30, 2021).

29. Meyers BM, Vogel A, Marotta P, et al. The Cost-Effectiveness of Lenvatinib in the Treatment of Advanced or Unresectable Hepatocellular Carcinoma from a Canadian Perspective. *Canadian journal of gastroenterology & hepatology* 2021; 2021: 8811018. 2021/03/09. DOI: 10.1155/2021/8811018.

For peer review only

Figure legends:

Figure 1: Tornado diagram of one-way deterministic sensitivity analysis (DSA) of LEN-TACE vs. LEN. The dashed line represents the threshold of willingness-to pay ($37,663/QALYs). ICER, incremental cost-effectiveness ratio; QALYs, quality-adjusted life-year.
Figure 2: Probabilistic sensitivity analysis scatter plot (1,000 iterations). An ellipse surrounds 95% of the estimates. WTP, willingness-to-pay; GDP, gross domestic product.
Figure 3: Cost-effectiveness acceptability curve of LEN-TACE vs. LEN. QALYs, quality-adjusted life-year.

For peer review only

For peer review only - http://bmjopen.bmj.com/site/about/guidelines.xhtml

Table 1: Model parameters.

Item	Base case	Range	Distribution	Source
**Costs (US $)**				
Lenvatinib per 120 mg (PATHEONINC)	499.71	399.77-499.71	Gamma	22
Regorafenib per 1120 mg (Bayer AG)	744.85	372.43- 744.85	Gamma	22
Tislelizumab per 100 mg (BeiGene)	223.63	178.91-223.63	Gamma	22
TACE per session	1929	1543-2315	Gamma	21
Hepatectomy	9022	7218-10827	Gamma	21
**Management of adverse events**				
Elevated ALT/AST	56.54	45.23-67.84	Gamma	20
Hypertension	35.46	28.36-42.55	Gamma	20
Hyperbilirubinemia	113.53	90.82-136.24	Gamma	15
Diarrhea	5.66	4.53-6.79	Gamma	19
Decreased weight	102.73	98.67- 120.63	Gamma	15
**BSC cost per cycle**	265.08	212.06-318.10	Gamma	22
**Follow-up and monitoring per month in PFS**	114	86-143	Gamma	22
**Follow-up and monitoring per month in PD**	210	157-262	Gamma	22
**Terminal care per patient**	1839	1519-2279	Gamma	22
**Utility values**				
PFS	0.76	0.61-0.91	Beta	25
PD	0.68	0.54-0.82	Beta	25
**Disutility of adverse events**				
Elevated ALT/AST	0	NA	NA	15
Hypertension	0.012	0.010-0.014	Beta	15
Hyperbilirubinemia	0	NA	NA	15
Diarrhea	0.047	0.016-0.077	Beta	19
Decreased weight	0.053	0.042-0.064	Beta	15
**LEN-TACE: Incidence of SAEs**				
Elevated ALT/AST	0.40	0.32-0.48	Beta	13
Hypertension	0.20	0.16-0.24	Beta	13
Hyperbilirubinemia	0.09	0.072-0.108	Beta	13
Diarrhea	0.05	0.04-0.06	Beta	13
Decreased weight	0.076	0.06-0.09	Beta	13
**LEN: Incidence of SAEs**				
Elevated ALT/AST	0.03	0.024-0.036	Beta	13
Hypertension	0.20	0.16-0.24	Beta	13
Hyperbilirubinemia	0.03	0.024-0.036	Beta	13
Diarrhea	0.04	0.032-0.048	Beta	13
Decreased weight	0.07	0.056-0.084	Beta	13
**Discount rate(%)**	5	0-8	Fixed in PSA	
**Proportion of receiving subsequent treatments in LEN-TACE**				
Hepatectomy	0.16	0.128-0.192	Beta	13
Regorafenib	0.22	0.176-0.264	Beta	13
Tislelizumab	0.43	0.344-0.516	Beta	13
BSC	0.19	0.152-0.228	Beta	13
**Proportion of receiving subsequent treatments in LEN**				
TACE	0.07	0.056-0.084	Beta	13
Regorafenib	0.25	0.20-0.30	Beta	13
Tislelizumab	0.51	0.408-0.612	Beta	13
BSC	0.17	0.136-0.204	Beta	13
**LEN treatment duration (months)**	15.0	8.0-15.0	Normal	13

BSC, best supportive care; PFS, progression-free survival; PD, progressed disease; PSA, probabilistic sensitivity analysis; LEN, lenvatinib; TACE, transarterial chemoembolization; SAEs, serious adverse events.

For peer review only

Table 2: Base-case and scenario analyses results.

	Treatment	Cost ($)	Δ cost	QALYs	Δ QALYs	ICER
**Base-case**						
	LEN-TACE	31,394	12,151	1.166	0.382	31,808
	LEN	19,243	NA	0.784	NA	NA
**Scenario**						
Weibull model	LEN-TACE	30,629	11,736	1.072	0.328	35,780
	LEN	18,893	NA	0.744	NA	NA
Royston/Parmar spline model	LEN-TACE	31,468	10,733	1.203	0.320	33,540
	LEN	20,735	NA	0.883	NA	NA

QALYs, quality-adjusted life years; ICER, incremental cost-effectiveness ratio; NA, not applicable; LEN, lenvatinib; TACE, transarterial chemoembolization.

For peer review only

For peer review only - http://bmjopen.bmj.com/site/about/guidelines.xhtml

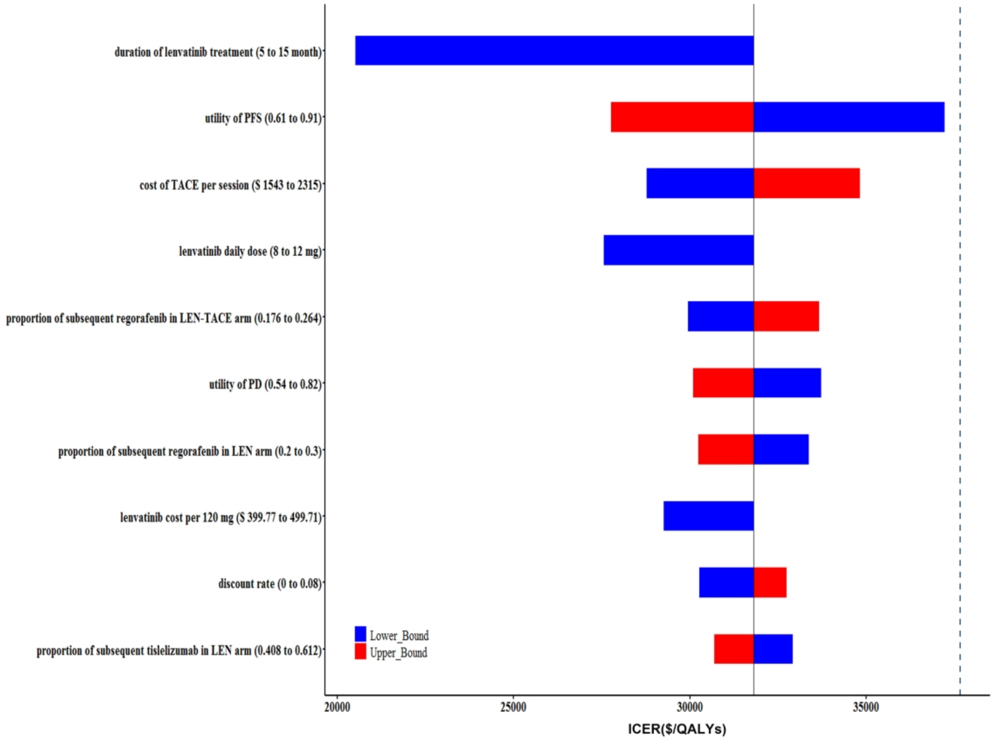

Figure 1: Tornado diagram of one-way deterministic sensitivity analysis (DSA) of LEN-TACE vs. LEN. The dashed line represents the threshold of willingness-to pay ($37,663/QALYs). ICER, incremental cost-effectiveness ratio; QALYs, quality-adjusted life-year.

129x96mm (600 x 600 DPI)

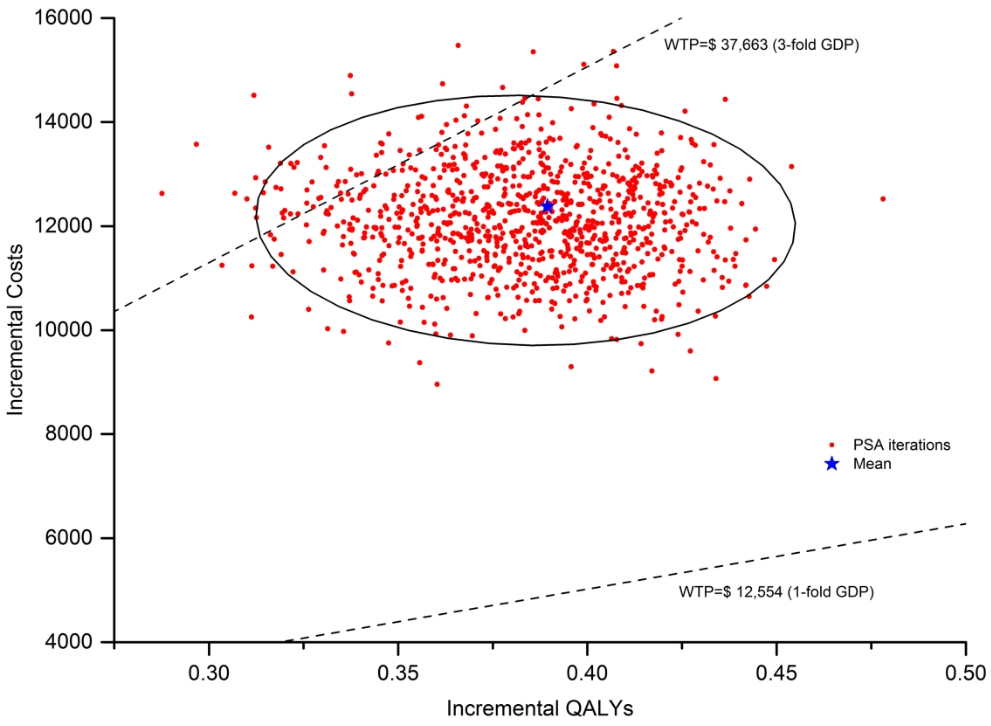

Figure 2: Probabilistic sensitivity analysis scatter plot (1,000 iterations). An ellipse surrounds 95% of the estimates. WTP, willingness-to-pay; GDP, gross domestic product.

231x168mm (300 x 300 DPI)

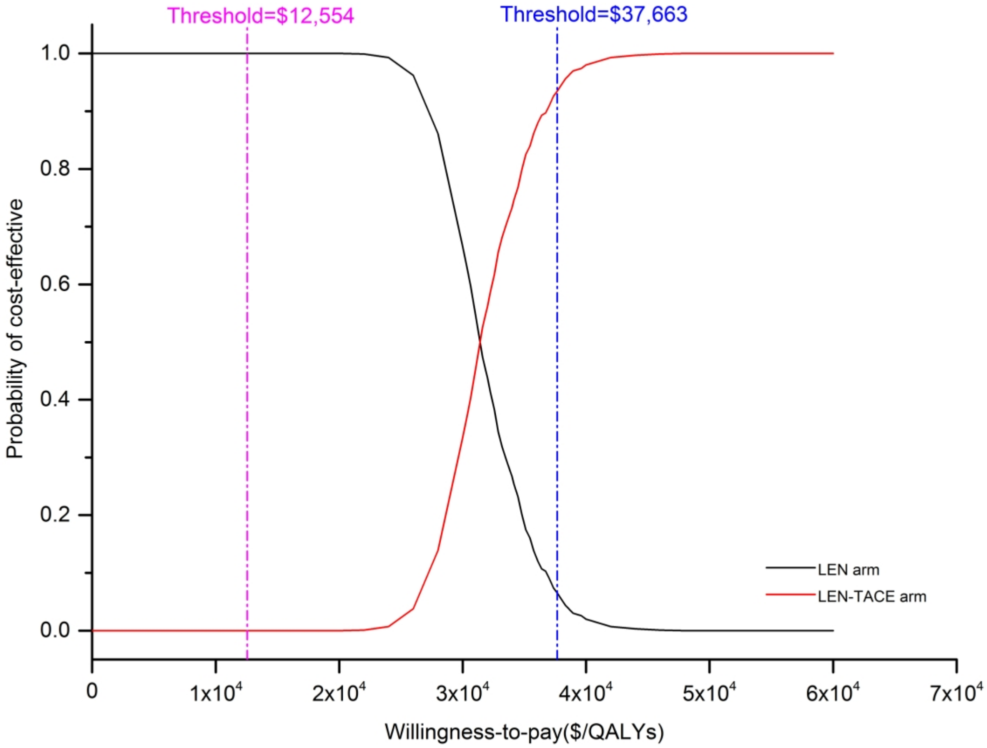

Figure 3: Cost-effectiveness acceptability curve of LEN-TACE vs. LEN. QALYs, quality-adjusted life-year.

227x174mm (300 x 300 DPI)

For peer review only

Supplementary Table S1: Fit parameters, AIC and BIC values for each model

			LEN-TACE				LEN			
**Index**	**Distribution**	**Parameter**	**Est**	**Se**	**AIC**	**BIC**	**Est**	**Se**	**AIC**	**BIC**
**PFS**	Log-logistic	shape	3.2270	0.2480	758.94*	765.21*	2.9320	0.2030	815.61*	821.86*
		scale	10.8880	0.4870			6.6220	0.3100		
	Gompertz	shape	0.1143	0.0152	806.42	812.70	0.0772	0.0145	879.20	885.45
		rate	0.0271	0.0048			0.0762	0.0099		
	Weibull	shape	2.1600	0.1540	775.03	781.30	1.7390	0.1050	841.79	848.04
		scale	13.7820	0.5960			8.8380	0.4360		
	Log-normal	meanlog	2.3910	0.0470	762.04	768.31	1.8878	0.0486	817.74	823.99
		sdlog	0.5513	0.0371			0.6067	0.0360		
	Gamma	shape	3.9865	0.4942	764.58	770.86	3.0411	0.3359	826.29	832.54
		rate	0.3246	0.0457			0.3888	0.0481		
	Exponential	rate	0.0671	0.0063	853.26	856.40	0.1198	0.0100	901.13	904.25
	Gen-gamma	mu	2.4161	0.0739	763.85	773.26	1.8818	0.0776	819.73	829.10
		sigma	0.5424	0.0421			0.6073	0.0365		
		Q	0.1055	0.2432			-0.0207	0.2082		
	Royston/Parmar spline model(scale=normal, 0 kont)	gamma0	-4.3370	0.2890	762.04	768.31	-3.1117	0.1961	817.74	823.99
		gamma1	1.8140	0.1220			1.6483	0.0978		
	Royston/Parmar spline model(scale=normal, 1 kont)	gamma0	-4.0295	0.5378	763.60	773.00	-3.0669	0.3074	819.71	829.08
		gamma1	1.6159	0.3179			1.6082	0.2342		
		gamma2	-0.0896	0.1345			-0.0128	0.0682		
	Royston/Parmar spline model(scale=normal, 2 kont)	gamma0	-3.4563	0.5592	762.03	774.57	-2.7900	0.3280	818.99	831.49
		gamma1	1.1548	0.3642			1.2590	0.2970		
		gamma2	-1.7142	0.8676			-0.7910	0.4710		
		gamma3	2.0204	1.0765			0.8290	0.5030		
	Royston/Parmar spline model(scale=normal, 3 kont)	gamma0	-3.3430	0.5660	763.68	779.36	-2.8390	0.3470	820.77	836.39
		gamma1	1.0410	0.3910			1.3370	0.3380		
		gamma2	-1.9230	1.9900			0.1340	1.4000		
		gamma3	1.7270	4.0660			-1.3300	2.5880		
		gamma4	0.2800	2.7960			1.3640	1.4860		
	Royston/Parmar spline model(scale=normal, 4 kont)	gamma0	-3.3579	0.5768	765.68	765.68	-2.9480	0.3750	821.45	840.20
		gamma1	1.0583	0.4067			1.5200	0.3900		
		gamma2	-1.3814	3.7579			1.9930	1.8950		
		gamma3	-0.3330	11.2152			-6.3750	5.798		
		gamma4	1.7765	11.3547			4.8450	5.7250		
		gamma5	0.0639	4.6610			-0.2470	1.9700		
	Royston/Parmar spline model(scale=normal, 5 kont)	gamma0	-3.3840	0.5890	766.75	766.75	-3.1760	0.4280	820.02	841.89
		gamma1	1.0830	0.4220			1.8950	0.4830		
		gamma2	1.3230	5.3950			4.0920	1.9290		
		gamma3	-12.2830	19.0650			-12.5980	6.4900		
		gamma4	17.4970	22.9360			15.0020	10.1850		
		gamma5	-10.8000	15.3220			-8.4620	7.7910		
		gamma6	5.0130	7.5430			2.6350	2.7250		
**OS**	Log-logistic	shape	3.3020	0.3160	582.22*	588.49*	2.9400	0.2380	723.78*	730.03*
		scale	17.9700	0.9050			11.7940	0.5960		
	Gompertz	shape	0.1391	0.0180	598.10	604.37	0.0811	0.0145	773.36	779.61
		rate	0.0073	0.0020			0.0298	0.0052		
	Weibull	shape	2.6100	0.2420	584.79	591.06	1.9440	0.1450	743.98	750.22
		scale	21.1940	0.9790			15.4080	0.7770		

Supplementary Table S1: Fit parameters, AIC and BIC values for each model

			LEN-TACE				LEN			
**Index**	**Distribution**	**Parameter**	**Est**	**Se**	**AIC**	**BIC**	**Est**	**Se**	**AIC**	**BIC**
	Log-normal	meanlog	2.8937	0.0569	585.65	591.92	2.4786	0.0538	726.80	733.05
		sdlog	0.5573	0.0470			0.6080	0.0431		
	Gamma	shape	4.4499	0.6680	583.19	589.47	3.3508	0.4286	732.93	739.18
		rate	0.2273	0.0408			0.2445	0.0367		
	Exponential	rate	0.0331	0.0038	654.31	657.45	0.0589	0.0058	799.13	802.25
	Gen-gamma	mu	2.9861	0.0734	585.14	594.55	2.4493	0.0893	728.63	738.00
		sigma	0.4595	0.0726			0.6167	0.0481		
		Q	0.5504	0.3377			-0.1120	0.2702		
	Royston/Parmar spline model(scale=normal, 0 kont)	gamma0	-5.1930	0.4100	585.65	591.92	-4.0770	0.2830	726.80	733.05
		gamma1	1.7950	0.1510			1.6450	0.1170		
	Royston/Parmar spline model(scale=normal, 1 kont)	gamma0	-3.9839	0.6927	583.99	593.40	-4.0452	0.5118	728.79	738.16
		gamma1	1.1578	0.3387			1.6253	0.2871		
		gamma2	-0.4984	0.2530			-0.0106	0.1433		
	Royston/Parmar spline model(scale=normal, 2 kont)	gamma0	-3.9100	0.7600	586.01	598.55	-3.3200	0.5010	723.12	735.61
		gamma1	1.1080	0.4000			1.0510	0.3100		
		gamma2	-0.5850	1.6860			-2.4310	0.8620		
		gamma3	0.2710	2.6460			3.0190	1.0760		
	Royston/Parmar spline model(scale=normal, 3 kont)	gamma0	-4.4770	0.9470	585.36	601.04	-3.5370	0.5570	721.47	737.09
		gamma1	1.4830	0.5240			1.2520	0.3600		
		gamma2	3.2720	2.2140			2.3380	2.5060		
		gamma3	-13.3810	8.0390			-8.6030	5.0560		
		gamma4	13.1590	8.3970			8.1020	3.3600		
	Royston/Parmar spline model(scale=normal, 4 kont)	gamma0	-4.4870	0.9800	587.42	587.42	-3.7100	0.6260	721.69	740.43
		gamma1	1.4910	0.5490			1.4040	0.4300		
		gamma2	2.6710	2.5600			1.6380	2.4290		
		gamma3	-5.4500	15.3950			-2.2890	8.3800		
		gamma4	-4.4210	25.1920			-6.9260	11.6520		
		gamma5	11.0260	16.5950			10.0550	6.3980		
	Royston/Parmar spline model(scale=normal, 5 kont)	gamma0	-4.3490	0.9860	589.24	589.24	-3.9170	0.6910	719.66	741.52
		gamma1	1.3990	0.5610			1.5940	0.4900		
		gamma2	0.2810	2.9780			4.2290	2.8250		
		gamma3	9.2650	16.6470			-15.6820	11.1400		
		gamma4	-23.8210	38.8820			25.5560	19.8710		
		gamma5	9.1510	45.3570			-32.2010	20.6110		
		gamma6	10.8110	27.9880			21.7010	10.4480		

*, best fitted model; PFS, progression-free survival; OS, overall survival; AIC, Akaike information criterion; BIC, Bayesian information criterion.

For peer review only

Supplementary Table S2: Validation of modeled PFS and OS data using internal and external data.

Curve	6-month (%)	12-month (%)	24-month (%)	36-month (%)
**PFS**				
LEN-TACE				
log-logistic	87.2	42.2	7.2	2.1
weibull	84.7	47.6	3.6	0.035
royston/parmar spline model (knot=5)	88.5	41.7	9.5	3.2
LAUNCH	88.2	39.2	11.4	NA
LEN				
log-logistic	57.2	14.9	2.2	0.7
weibull	60.0	18.2	0.3	0.001
royston/parmar spline model (knot=5)	56.9	14.2	2.9	1.0
LAUNCH	54.8	14.3	2.5	NA
**OS**				
LEN-TACE				
log-logistic	97.4	79.1	27.8	9.2
weibull	96.4	79.7	25.1	1.9
royston/parmar spline model (knot=5)	96.7	80.9	28.3	12.1
Chen Song et al, 2022	84.9	63.5	18.5	9.5
LAUNCH	95.9	81.5	26.1	NA
LEN				
log-logistic	87.9	48.7	11.0	3.6
weibull	85.2	54.1	9.4	0.6
royston/parmar spline model (knot=2)	89.8	45.0	16.2	8.0
REFLECT	82.1	55.0	29.8	13.0
LAUNCH	87.7	47.0	17.8	NA

PFS, progression-free survival; OS, overall survival; NA, not applicable.

For peer review only

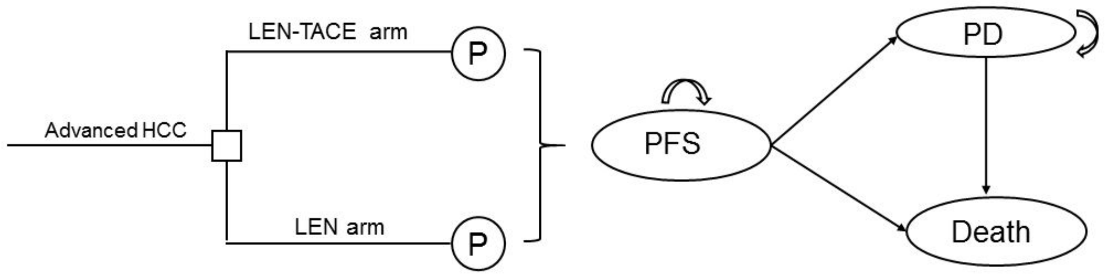

Supplementary Figure S1: Model structure of a decision tree combining the partitioned survival model. (HCC, hepatocellular carcinoma; LEN, lenvatinib; TACE, transarterial chemoembolization; PFS, progression-free survival; PD, progressed survival).

For peer review only

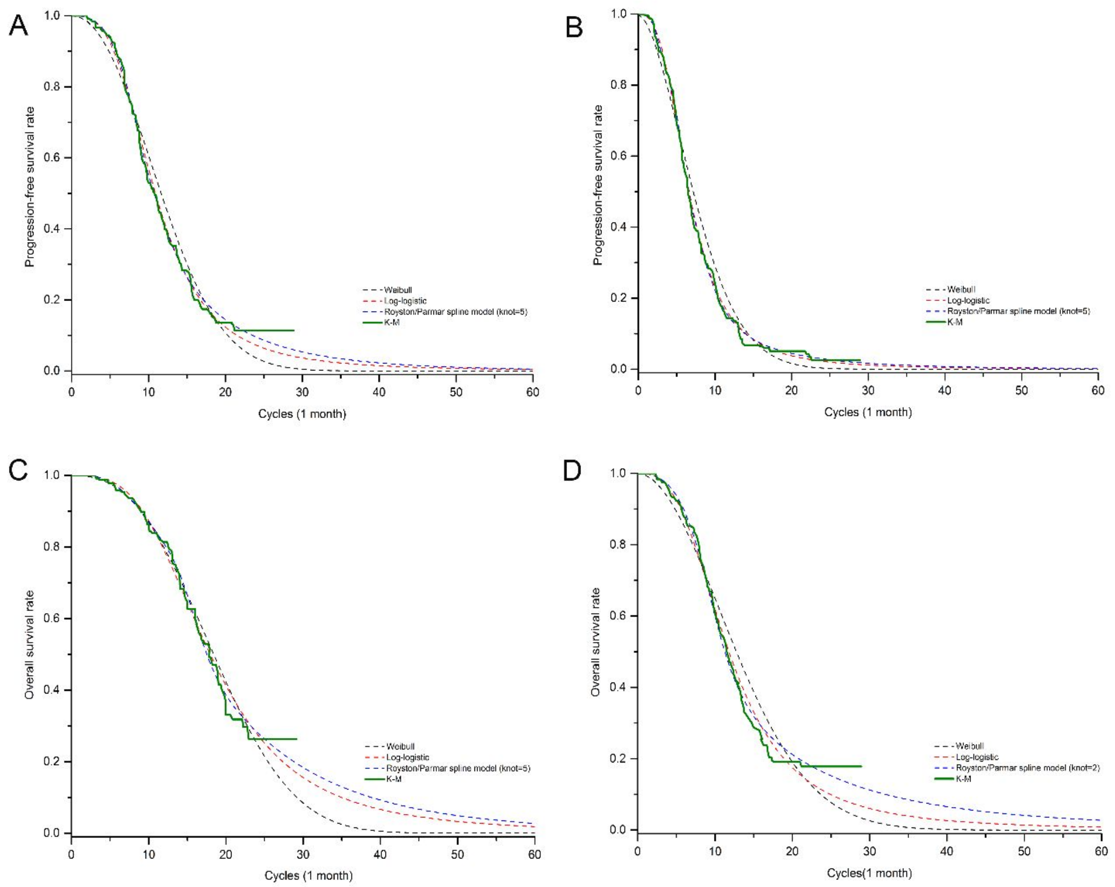

Supplementary Figure S2: The exploration and fitting of PFS and OS curves in the partitioned survival model. (A) PFS of LEN-TACE arm, (B) PFS of LEN arm, (C) OS of LEN-TACE arm, (D) OS of LEN arm.

eview only

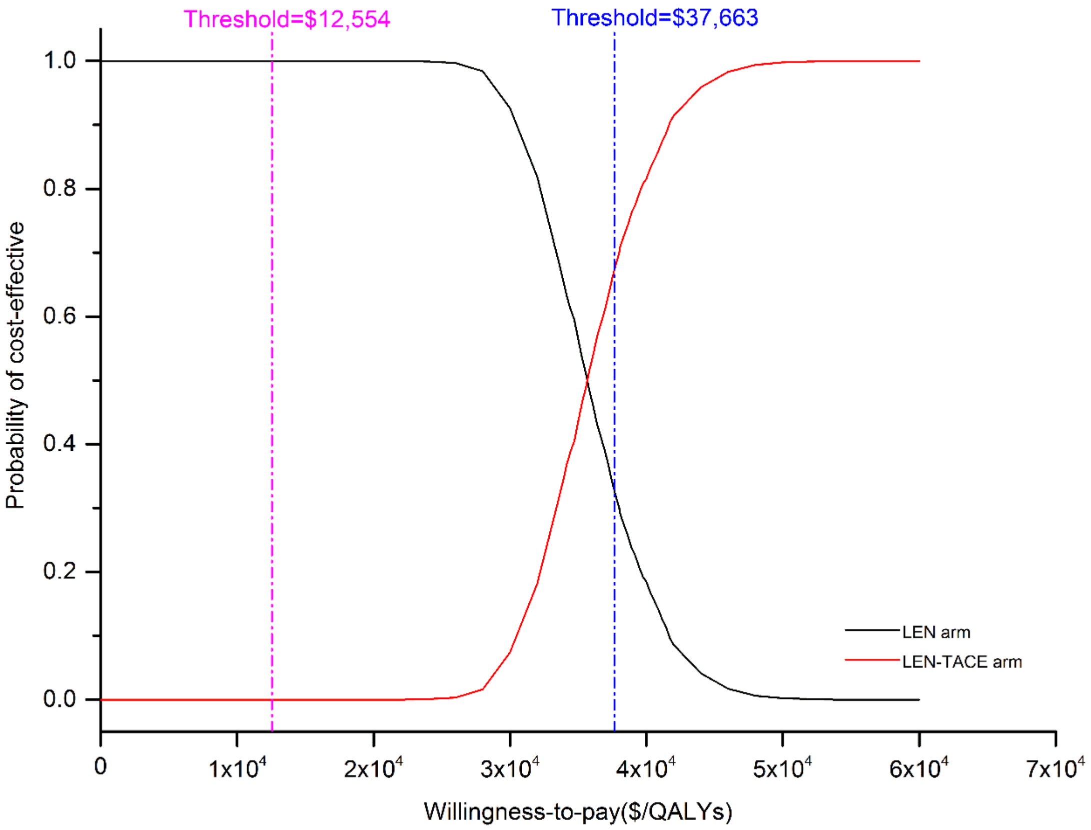

Supplementary Figure S3: Cost-effectiveness acceptability curve of LEN-TACE vs. LEN adopting Weibull distribution model. QALYs, quality-adjusted life-year.

review only

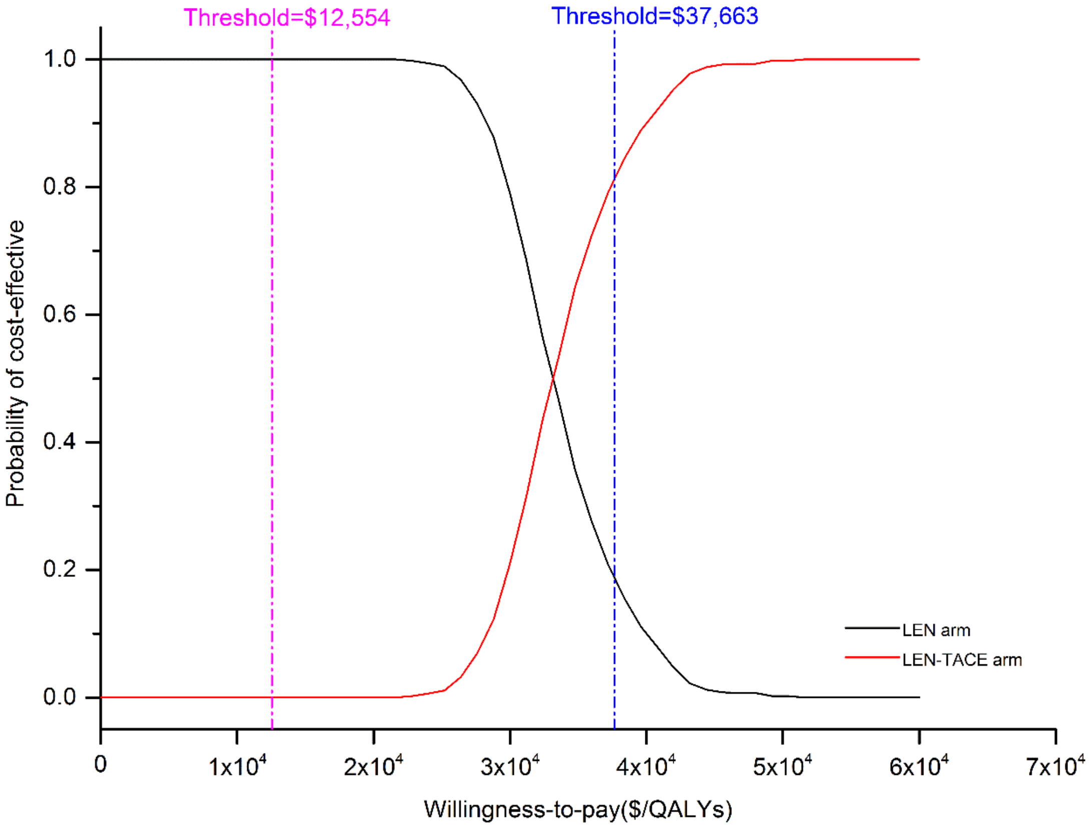

Supplementary Figure S4: Cost-effectiveness acceptability curve of LEN-TACE vs. LEN adopting Royston/Parmar spline model. QALYs, quality-adjusted life-year.

review only

CHEERS 2022 checklist			
Section/topic	Item no.	Guidance for reporting	Reported in section
Title	1	Identify the study as an economic evaluation and specify the interventions being compared.	title
Abstract			
Abstract	2	Provide a structured summary that highlights context, key methods, results, and alternative analyses.	abstract
Introduction			
Background and objectives	3	Give the context for the study, the study question, and its practical relevance for decision making in policy or practice.	Introduction
Methods			
Health economic analysis plan	4	Indicate whether a health economic analysis plan was developed and where available.	Not Applicable
Study population	5	Describe characteristics of the study population (such as age range, demographics, socioeconomic, or clinical characteristics).	Model structure and outcomes
Setting and location	6	Provide relevant contextual information that may influence findings.	Not Reported
Comparators	7	Describe the interventions or strategies being compared and why chosen.	Model Structure and Outcomes
Perspective	8	State the perspective(s) adopted by the study and why chosen.	Model Structure and Outcomes
Time horizon	9	State the time horizon for the study and why appropriate.	Model structure and outcomes
Discount rate	10	Report the discount rate(s) and reason chosen.	Model structure and outcomes
Selection of outcomes	11	Describe what outcomes were used as the measure(s) of benefit(s) and harm(s).	Model structure and outcomes
Measurement of outcomes	12	Describe how outcomes used to capture benefit(s) and harm(s) were measured.	Model structure and outcomes
Valuation of outcomes	13	Describe the population and methods used to measure and value outcomes.	Cost and utility
Measurement and valuation of resources and costs	14	Describe how costs were valued.	Cost and utility
Currency, price date, and conversion	15	Report the dates of the estimated resource quantities and unit costs, plus the currency and year of conversion.	Model structure and outcomes
Rationale and description of model	16	If modeling is used, describe in detail and why used. Report if the model is publicly available and where it can be accessed.	Clinical data and transition probabilities

For peer review only

Analytics and assumptions	17	Describe any methods for analysing or statistically transforming data, any extrapolation methods, and approaches for validating any model used.	Clinical data and transition probabilities and Model validation
Characterizing heterogeneity	18	Describe any methods used for estimating how the results of the study vary for subgroups.	Sensitivity Analysis and Scenario Analysis
Characterizing distributional effects	19	Describe how impacts are distributed across different individuals or adjustments made to reflect priority populations.	Sensitivity Analysis and Scenario Analysis
Characterizing uncertainty	20	Describe methods to characterize any sources of uncertainty in the analysis.	Sensitivity Analysis and Scenario Analysis
Approach to engagement with patients and others affected by the study	21	Describe any approaches to engage patients or service recipients, the general public, communities, or stakeholders (such as clinicians or payers) in the design of the study.	Not Applicable
Results			
Study parameters	22	Report all analytic inputs (such as values, ranges, references) including uncertainty or distributional assumptions.	Table 1, and Supplementary Table S1
Summary of main results	23	Report the mean values for the main categories of costs and outcomes of interest and summarize them in the most appropriate overall measure.	Table 2
Effect of uncertainty	24	Describe how uncertainty about analytic judgments, inputs, or projections affect findings. Report the effect of choice of discount rate and time horizon, if applicable.	Figure 1,2, 3, 4 and Supplementary Figure S2, S3
Effect of engagement with patients and others affected by the study	25	Report on any difference patient/service recipient, general public, community, or stakeholder involvement made to the approach or findings of the study	Not Reported
Discussion			
Study findings, limitations, generalizability, and current knowledge	26	Report key findings, limitations, ethical or equity considerations not captured, and how these could affect patients, policy, or practice.	discussion
Other relevant information			
Source of funding	27	Describe how the study was funded and any role of the funder in the identification, design,	Funding

For peer review only

		conduct, and reporting of the analysis	
Conflicts of interest	28	Report authors conflicts of interest according to journal or International Committee of Medical Journal Editors requirements.	Competing interests
From: Husereau D, Drummond M, Augustovski F, et al. Consolidated Health Economic Evaluation Reporting Standards 2022 (CHEERS 2022) Explanation and Elaboration: A Report of the ISPOR CHEERS II Good Practices Task Force. Value Health 2022;25. doi:10.1016/j.jval.2021.10.008			

For peer review only

Cost-effectiveness analysis of adding transarterial chemoembolization to lenvatinib as first-line treatment for advanced hepatocellular carcinoma in China

Journal:	*BMJ Open*
Manuscript ID	bmjopen-2023-074245.R1
Article Type:	Original research
Date Submitted by the Author:	16-Aug-2023
Complete List of Authors:	Li, Wei; Huazhong University of Science and Technology Tongji Medical College, Department of Pharmacy, Maternal and Child Health Hospital of Hubei Province<br>Wan, Li; Huazhong University of Science and Technology Tongji Medical College, Department of Pharmacy, Maternal and Child Health Hospital of Hubei Province
**Primary Subject Heading**:	Health economics
Secondary Subject Heading:	Health economics, Oncology, Pharmacology and therapeutics, Public health
Keywords:	HEALTH ECONOMICS, Health economics < HEALTH SERVICES ADMINISTRATION & MANAGEMENT, Hepatology < INTERNAL MEDICINE, Clinical Decision-Making, Decision Making

SCHOLARONE™
Manuscripts

I, the Submitting Author has the right to grant and does grant on behalf of all authors of the Work (as defined in the below author licence), an exclusive licence and/or a non-exclusive licence for contributions from authors who are: i) UK Crown employees; ii) where BMJ has agreed a CC-BY licence shall apply, and/or iii) in accordance with the terms applicable for US Federal Government officers or employees acting as part of their official duties; on a worldwide, perpetual, irrevocable, royalty-free basis to BMJ Publishing Group Ltd ("BMJ") its licensees and where the relevant Journal is co-owned by BMJ to the co-owners of the Journal, to publish the Work in this journal and any other BMJ products and to exploit all rights, as set out in our licence.

The Submitting Author accepts and understands that any supply made under these terms is made by BMJ to the Submitting Author unless you are acting as an employee on behalf of your employer or a postgraduate student of an affiliated institution which is paying any applicable article publishing charge ("APC") for Open Access articles. Where the Submitting Author wishes to make the Work available on an Open Access basis (and intends to pay the relevant APC), the terms of reuse of such Open Access shall be governed by a Creative Commons licence – details of these licences and which Creative Commons licence will apply to this Work are set out in our licence referred to above.

Other than as permitted in any relevant BMJ Author's Self Archiving Policies, I confirm this Work has not been accepted for publication elsewhere, is not being considered for publication elsewhere and does not duplicate material already published. I confirm all authors consent to publication of this Work and authorise the granting of this licence.

For peer review only

Cost-effectiveness analysis of adding transarterial chemoembolization to lenvatinib as first-line treatment for advanced hepatocellular carcinoma in China

Wei Li, and Li Wan

Department of Pharmacy, Maternal and Child Health Hospital of Hubei Province, Tongji Medical College, Huazhong University of Science and Technology, Wuhan 430070, China

Correspondence to:

Wei Li

Department of Pharmacy, Maternal and Child Health Hospital of Hubei Province, Tongji Medical College, Huazhong University of Science and Technology, Wuhan 430070, China

liwei1988hust@126.com

For peer review only

Abstract

Objective The objective of this study was to evaluate the comparative cost-effectiveness of lenvatinib (LEN) plus transarterial chemoembolization (TACE) [LEN-TACE] and LEN alone to treat advanced hepatocellular carcinoma (HCC) from the perspective of the Chinese healthcare system.
Design A three-state partitioned survival model using clinical survival data from a phase III LAUNCH trial, a 5-year time horizon for costs and quality-adjusted life-years (QALYs) was constructed to analyse the cost-effectiveness of LEN-TACE. Clinical inputs were extracted from the LAUNCH trial, with outcomes extrapolated using standard and flexible parametric survival models. Costs and utilities derived from published literature were discounted at an annual rate of 5%. Sensitivity analyses, and scenario analyses were conducted to test the robustness of the model.
Setting The Chinese healthcare system perspective.
Participants A hypothetical Chinese cohort of patients with advanced HCC.
Interventions Transarterial chemoembolization plus lenvatinib versus lenvatinib.
Primary outcome measure Costs, QALYs, incremental cost-effectiveness ratio (ICER).
Results: Base-case analysis revealed that LEN-TACE would be cost-effective in China at the willingness-to-pay (WTP) threshold of $37,663 per QALYs, with improved effectiveness of 0.382 QALYs and additional cost of $12,151 (ICER: $31,808 per QALY). The probabilistic sensitivity analysis suggested that LEN-TACE had a 93.5% probability of cost-effectiveness at WTP threshold of 3 times GDP per capital ($37,663). One-way deterministic sensitivity analysis indicated that the duration of lenvatinib treatment in both two arms, utility of PFS, and the cost of TACE had a greater impact on the stability of ICER values. Scenario analyses results were in line with base-case analysis.
Conclusions: LEN-TACE might be a cost-effective strategy compared with LEN for the first-line treatment of patients with advanced HCC in China.

Strengths and limitations of this study

- The partitioned survival model acquires the proportion of patients in different health states directly from the survival curves, without too many assumptions and is commonly applied in economic evaluations of advanced or metastatic cancer.
- Both standard and flexible parametric survival models are used to predict the long term survival outcomes in this cost-effectiveness analysis.
- External data accessed through reconstructing the survival curves of previously clinical trials on HCC is used to verify our model choice.
- In this model, the proportions of patients receiving subsequent anticancer treatment originated from clinical trial data, which might not true represent the prevalence of subsequent anticancer strategy used in real-world practice.
- We do not conduct the subgroup analysis due to a short of subgroup survival data.

For peer review only - http://bmjopen.bmj.com/site/about/guidelines.xhtml

Introduction

Hepatocellular carcinoma (HCC) is the six-most commonly diagnosed cancer and ranks third in cancer-related mortality worldwide.[1] However, this situation is even worse in China, where it is the fifth most commonly diagnosed cancer and the secondary leading cause of cancer-related death.[2] It is estimated that more than 70% of HCC patients have advanced cancer at the time of diagnosis, which limits the feasibility of radical surgical treatment in advanced cases.[3,4] Therefore, targeted systemic therapies have become a ray of hope for advanced HCC patients. Lenvatinib (LEN), an oral multiple receptor tyrosine kinase inhibitor, noninferior to sorafenib, is currently recommended as first-line treatments for advanced HCC.[5,6] Although the median overall survival (OS) for Chinese subgroup population was longer than that of the intention-to-treat (ITT) population (15.0 vs. 13.6 months), the efficacy of LEN is still unsatisfactory.[7] To further prolong the overall survival, much endeavors have been made, such as the combination of LEN with other treatments.

Transarterial chemoembolization (TACE) can effectively reduce tumor burden by targeting intrahepatic tumors, thus may enhance the outcomes in patients with advanced HCC.[8] While, LEN is more likely to be effective in patients with lower tumor burden.[9] In terms of mechanism of action, the combination of LEN and TACE (LEN-TACE) has a synergistic effect. Recently, a series of observational studies indicate that the combination of LEN and TACE brings favorable survival outcomes.[10-12] However, these observation studies only provide limited supporting evidence and need to be further verified by large-scale clinical trial.

Nowadays, a Chinese-patient-based phase III trial, LAUNCH (NCT03905967), demonstrates that LEN-TACE prolongs median OS by a significant 6.3 months, compared with LEN monotherapy in the ITT population.[13] Additionally, LEN-TACE shows a statistically significant improvement in progression-free survival (PFS) time, with a median improvement in PFS of 4.2 months.[13] Although the remarkable results of this trial raises hope for patients extend survival, higher medical costs generated by combination therapy may impose a heavy socioeconomic burden on patients and the healthcare system, especially in resource-limiting settings like China. At present, economic evaluations of LEN in the area of HCC mainly focus on the comparision of intervention regimes, such as the LEN vs. LEN similar drugs (sorafenib, donafenib), LEN vs. sintilimab plus bevacizumab.[14-16] There is no cost-effectiveness evaluation of LEN-TACE strategy in patients with advanced HCC from the perspective of Chinese healthcare system. We thus compare the cost-effectiveness of the two strategies to treat advanced HCC by using model data from LAUNCH. These findings provide evidence for use by HCC patients and the physicians treating them, as well as health policymakers.

Methods

Model Structure and Outcomes

A partitioned survival model (PSM), including three mutually exclusive disease-related health states: progression-free (PFS), progressed disease (PD), and death, was constructed in TreeAge Pro 2020 (TreeAge Software, Williamstown, MA) to simulate the cohort of patients in LAUNCH trial.[13] The model cycle length was a treatment cycle (1 month). The time horizon was set as 5 years, with 99% of people dying. This analysis was performed from the

For peer review only - http://bmjopen.bmj.com/site/about/guidelines.xhtml

perspective of Chinese healthcare system. Our target population was patient with primary advanced HCC receiving first-line treatments in China. These patients underwent lenvatinib (LEN) alone or plus TACE (LEN-TACE) during the simulated time. The tree diagram and bubble diagram were illustrated in **Supplementary Figure S1**. It was hypothesized that the patient cohort entered the model in the PFS state and then either occupied in the same state or moved to the other states according to transition probabilities during each model cycle. The initial age of the simulated cohort population was set to 55 years old, which was consistent with the average age of the LAUNCH trial.[13]

The primary model outcomes were the corresponding total costs of two therapeutic regimens, quality-adjusted life years (QALYs), and the incremental cost-effectiveness ratios (ICERs). Both costs and utility values were discounted at 5% annually for base-case analysis, according to guideline for health economic evaluations in China.[17] All costs were converted into 2021 US dollars (US 1$ = CNY ¥6.45). Three-fold of the per capita gross domestic product (GDP) of China in 2021 (US$37,663/QALYs) was used as the willingness-to-pay (WTP) threshold to evaluate the cost-effectiveness of the two competing strategies.[17]

Clinical Data and Model Probabilities

The clinical efficacy and safety data of LAUNCH trial, which was conducted at 12 hospitals in China, were used to explore the cost-effectiveness of lenvatinib with or without TACE for advanced HCC.[13] The PFS and OS data were extracted from the published K-M curves by using GetData Graph Digitizer software (version 2.26) and then was utilized to reconstruct the individual patient data (IPD) according to Guyot's method.[18] To extrapolate survival outcomes outside the observation period, different parameter distributions (exponential, weibull, gompertz, log-logistic, log-normal, gamma, gen-gamma and royston/parmar spline model) were employed to fit the reconstructed IPD. The choice of survival model to use was based on statistical goodness of fit (using the Akaike's information criterion (AIC) and the Bayesian information criterion (BIC)), visual inspection and clinical plausibility of the extrapolations. Finally, the log-logistics model had the best AIC and BIC was used for both arms for PFS and OS in base-case analysis (**see Supplementary Table S1**). We also provided the exploration and fitting of PFS and OS curves for visual inspection (**see Supplementary Figure S2**). The choice of log-logistic model as survival distribution for extrapolation in base-case analysis was consistency with previous findings of extrapolation in advanced HCC.[19] The log-logistic model used for OS in LEN-TACE arm estimated a 3-year survival rate of 9.2% compared with that of 9.5%, reported by a retrospective study (median follow-up time, 27 months) in China.[20] Although the log-logistic model used for OS in LEN arm predicted a 3-year survival rate of 3.6%, which was not consistent with that of 13% reported in REFLECT trial, we thought that this model exhibited a reasonable fit to the observed data.[21] The reasons were as follows: (a) There were some differences in baseline patient characteristics between LAUNCH and REFLECT trial, especially in α-fetoprotein and Barcelona Clinic Liver Cancer stage. (b) The higher α-fetoprotein level and more advanced Barcelona Clinic Liver Cancer stage in LAUNCH trial might be associated with lower OS survival rate. The impact of selecting alternative survival model was investigated in scenario analyses.

Cost and Utility

Only direct medical costs, including the first-line and subsequent treatment cost, monitoring cost, hepatectomy cost, TACE cost, management of serious adverse events (SAEs, grade 3 to

For peer review only - http://bmjopen.bmj.com/site/about/guidelines.xhtml

4), follow-up cost, and cost of terminal care in end of life, were calculated from the perspective of the Chinese healthcare system. It should be noted that, follow-up costs included CT examination, urinalysis, blood test, and blood biochemical examination; monitoring costs included diagnosis fee, nursing fee, injection fee, and bed fee, more details were presented in **Table 1**. Based on the LAUNCH trial, the median number of TACE sessions per patient was 3 (range, 1-6 sessions), once every 8 weeks.[13] All costs were derived from previously published literature.[22-25] To calculate the costs in base-case analysis, we made the following assumptions in this model:

a) We assumed that the average weight of a patient was 60 Kg.
b) We did not consider any cause of dose reduction or interruption of lenvatinib in base-case analysis. Patients were assumed to receive full dose lenvatinib (body weight ≥ 60 Kg, 12mg daily; body weight < 60 Kg, 8mg daily) until disease progression or unacceptable toxicity.
c) In line with the LAUNCH trial protocol, patients after progression or discontinuation would receive subsequent treatments, such as, curative surgical resection, tyrosine kinase inhibitor (TKI) therapy, programmed cell death protein-1 (PD-1) inhibitor and so on. The selection of specific TKI and PD-1inhibitor as subsequent treatment was based on the guideline of China.[26] For simplification, we assumed that patients in LEN-TACE arm would receive one of the following subsequent treatments: hepatectomy, regorafenib (160 mg/day, 3 weeks of medications, then discontinuing for 1 week), tislelizumab (200mg/day, q3w), best supportive care (BSC); Patients in LEN arm would receive one of the following subsequent treatments: regorafenib (160 mg/day, 3 weeks of medications, then discontinuing for 1 week), tislelizumab (200mg/day, q3w), TACE, BSC. The proportion of patients receiving subsequent therapy was derived from the LAUNCH trial.
d) To better reflect the cost of real-world clinical practice, the duration of treatment in PFS state was considered. The cost for lenvatinib was charged based on the upper limit of the treatment duration, as per the LAUNCH trial. In addition, a 2-year maximum treatment duration of tislelizumab was taken into consideration based on previous study.[27]
e) Only grade 3 or 4 SAEs with an incidence of >5% were considered, including elevated alanine aminotransferase (ALT)/ aspartate aminotransferase (AST), hypertension, decreased weight, diarrhea, and hyperbilirubinemia. The 3-4 SAEs-related costs were calculated once in the first cycle by multiplying the incidence of the SAEs by the costs of managing the SAEs per event.
f) Given that the subsequent treatment information was limited from the LAUNCH trial records. The grade 3-4 SAEs-related costs for subsequent treatment were ignored.
g) We assumed that all the patients received terminal care three months before they died in the base-case analysis.

Health state utility values were derived from previously published cost-effectiveness studies regarding Chinese patients with unresectable HCC, and the values were set at 0.76 for the PFS, 0.68 for PD state respectively.[28] Considering that the occurrence of adverse events might have impact on patients' health-related quality of life. This present study also considered the disutility due to adverse events, including elevated ALT/AST, hypertension, decreased weight, diarrhea, and hyperbilirubinemia, which were obtained from previous researches.[15,22] The details were listed in **Table 2**.

Sensitivity Analysis and Scenario Analysis

One-way deterministic (DSA) and probabilistic sensitivity analysis (PSA) were performed to examine the robustness of model outcomes. To identify factors that had substantial impact on

For peer review only - http://bmjopen.bmj.com/site/about/guidelines.xhtml

ICER, all variables varied over the plausible ranges, which were obtained from their corresponding confidence intervals (CIs) or ±20% of the base-case values (**Table 1)**. It should be noted that a bodyweight less than 60 Kg associated with lower lenvatinib dose (8 mg daily), resulting in the difference in treatment costs should not be ignored. Hence, we assumed a HCC patient weighted less than 60 Kg (lenvatinib, 8 mg daily) to investigate the impact on model outcomes. The results of DSA were graphed in the tornado diagram. PSA was performed to determine the effects of uncertainty in all model parameters simultaneously varied with prespecified distributions via 1,000 Monte Carlo simulations, which were illustrated in scatter plot and cost-effectiveness acceptability curves (CEACs). Specifically, utility values, probabilities or proportions were assigned beta distributions, costs and treatment duration were apportioned to gamma distribution and normal distributions, respectively. The key parameters input our model were shown in **Table 1 and Supplementary Table S1**.

We also conducted two scenario analyses to explore the impact of different key model setting and assumptions on the economic outcomes. To eradicate the uncertainty caused by the extrapolation of the survival curves, we used different parametric distribution survival models (i.e. weibull and royston/parmar spline model) to estimate the ICER. The weibull distribution obtained lower survival benefit compared to that of other distributions in this study, so it was suitable to examine the model stability under extreme conditions. The parameter values of weibull distribution and royston/parmar spline model for PFS and OS curves were exhibited in **Supplementary Table S1**.

Patient and public involvement

No patients or public were involved in the study.

Results

Base-Case Analysis

In our base-case analysis, LEN-TACE cost $31,394 and yielded 1.166 QALYs, whereas LEN cost $19,243 and yielded 0.784 QALYs (**see Table 2**). The ICER of LEN-TACE vs. LEN was $31,808 per QALY, which was below the WTP threshold ($37,663/QALYs), demonstrating that the LEN-TACE was a cost- effective treatment option for advanced HCC.

Sensitivity Analysis and Scenario Analysis

DSA result indicated that the model was most sensitive to the duration of lenvatinib treatment in both two arms, utility of PFS, and the cost of TACE. We only presented the top 10 sensitive factors in **Figure 1**. All parameters fluctuated within the range in the DSA did not bring the ICER values surpass the WTP threshold ($37,663/QALYs). When the duration of lenvatinib treatment in both two arms increased from 5 to 12 months in this model, the ICER went up from $20,508 to $31,808 per QALYs correspondingly. For patient with a bodyweight <60 Kg, namely the lenvatinib daily dose reduced from 12 to 8 mg, the ICER dropped from $31,808 to $27,559 per QALYs. In other words, for patients with lower bodyweight, LEN-TACE had more favorable economic outcomes in comparison with LEN. As shown in **Figure 2**, most of scatter points were between the line of 1- and 3-fold GDP. The CEACs also indicated that the probability of LEN-TACE being cost-effective was about

For peer review only - http://bmjopen.bmj.com/site/about/guidelines.xhtml

93.5% at the threshold of $37,663/QALYs, which was consistent with those of base-case analysis outcomes (**Figure 3**).

Assuming alternative weibull and royston/parmar spline model would lead to the slight increase of ICER values ($35,780, 33,540 vs. $31,808) compared with that of log-logistic model, respectively (**Table 2**). Overall, the results of scenario analyses were congruous with the conclusions of the base-case analysis, confirming the robustness of our model outcomes. The CEACs of scenario analyses were given in **Supplementary Figure S3-4**.

Model Validation

External data accessed through reconstructing the survival curves of previously clinical trials on HCC was used to verify our model choice.[20,21] Validation results for modeled PFS and OS data were presented in **Supplementary Table S2 and Supplementary Figure S2**. The 6, 12, 24, 36-month survival rate estimated by best-fitting distribution and alternative models compared with the results that observed in LAUNCH trial and external data were presented in **Supplementary Table S2 and Supplementary Figure S2**.

Discussion

To our knowledge, this is the first study to assess the economic outcomes of adding TACE to lenvatinib as a first-line treatment for advanced HCC in China. The findings of this study suggested that the addition of TACE to lenvatinib, as an alternative treatment option for Chinese patients with advanced HCC, might shed light on a potentially cost-effective practice-changing opportunity for clinicians, researchers, and policymakers in the future.

In this study, we demonstrated that LEN-TACE might be a cost-saving treatment option for advanced HCC patients, with ICER versus LEN of $31,808 per QALYs. PSA results suggested that the probability of LEN-TACE being cost-effective was 93.5% at the WTP threshold of $37,663/QALYs, which proved the robustness of the base-case analysis results. When choosing alternative survival models, such as weibull and royston/parmar spline model, the conclusion of LEN-TACE strategy being cost-effective was unchanged with a probability of 68.4% and 83.0%, respectively (**Supplementary Figure S3-4**). The DSA results revealed that the ICER was most sensitive to the duration of lenvatinib treatment in both two arms, utility of PFS, and the cost of TACE. However, none of parameter variation could lead to an inversion of this economic evaluation results. It also should be noted that the utility value of PFS varying within a relatively wide range (0.61-0.91) would lead to a very close to the threshold of WTP. However, if we input a narrowed PFS utility value variation range (0.73-0.76) as reported by NICE single technology appraisal in our model, the obtained ICER ($31,804-32,759/QALYs) was significantly below the threshold line.[29] In summary, the outcomes of this economic evaluation model were reliable.

A number of existing limitations in this study should be noted. First, due to the lack of utility based on only local populations, the EQ-5D utilities of the PFS and PD states deriving from previously published cost-effectiveness analysis considering advanced HCC were used in this model, which may bring bias. We will update our model outcomes when these data are available in China. Second, the health outcomes beyond the follow-up period were estimated by parametric distributions, and the selection of the best fit distribution largely depend on the long follow-up external data. Inappropriate choice of parameter models would result in overestimation or underestimation of survival rate, which in turn, brought bias to the

For peer review only - http://bmjopen.bmj.com/site/about/guidelines.xhtml

economic evaluation. Third, the proportions of patients receiving subsequent anticancer treatment originated from clinical trial data, which might not true represent the prevalence of subsequent anticancer strategy used in real-world practice. Fourth, we did not conduct the subgroup analysis due to a short of subgroup PFS and OS data. We will continue to investigate subgroups patients in the future when more data on subgroup populations become available.

Conclusions

The findings of this economic evaluation suggest that the combination of lenvatinib and TACE is likely to be a cost-effectiveness treatment option for Chinese patients with advanced HCC under the WTP threshold of $37,663 in China. In the future, further long-term follow-up data and real-world data are needed to verify the robustness of model outcomes.

Acknowledgements

None.

Contributors

WL contributed to the study concepts and design, analysis and interpretation of the data, drafting and revising of the paper; LW contributed to the review of the paper and supervision; all authors approved of final manuscript; all authors agree to be accountable for all aspects of the work.

Funding

This work was supported by the Maternal and Child Health Hospital of Hubei Province Research Project (No. 2021SFYM030).

Competing interests

The authors declare that there is no conflict of interest.

Patient consent for publication

Not required.

Ethics approval

Ethical approval was not necessary, because our economic evaluation is based on a mathematical model analysis, and does not contain any studies with human participants or animals performed.

Provenance and peer review

Not commissioned; externally peer reviewed.

For peer review only

Data availability statement

Data are available upon reasonable request.

Supplemental material

Supplemental material for this article is available online.

References

1. Sung H, Ferlay J, Siegel RL, et al. Global Cancer Statistics 2020: GLOBOCAN Estimates of Incidence and Mortality Worldwide for 36 Cancers in 185 Countries. *CA Cancer J Clin* 2021; 71: 209-249. 2021/02/05. DOI: 10.3322/caac.21660.

2. International Agency for Research on Cancer. (2020). All Cancer Source: Globocan 2020. Available online at: https://gco.iarc.fr/ (accessed September 8, 2021).

3. Llovet JM and Bruix J. Novel advancements in the management of hepatocellular carcinoma in 2008. *J Hepatol* 2008; 48 Suppl 1: S20-37. 2008/02/29. DOI: 10.1016/j.jhep.2008.01.022.

4. Park JW, Chen M, Colombo M, et al. Global patterns of hepatocellular carcinoma management from diagnosis to death: the BRIDGE Study. *Liver Int* 2015; 35: 2155-2166. 2015/03/11. DOI: 10.1111/liv.12818.

5. Benson AB, D'Angelica MI, Abbott DE, et al. Hepatobiliary Cancers, Version 2.2021, NCCN Clinical Practice Guidelines in Oncology. *J Natl Compr Canc Netw* 2021; 19: 541-565. 2021/05/25. DOI: 10.6004/jnccn.2021.0022.

6. Chen LT, Martinelli E, Cheng AL, et al. Pan-Asian adapted ESMO Clinical Practice Guidelines for the management of patients with intermediate and advanced/relapsed hepatocellular carcinoma: a TOS-ESMO initiative endorsed by CSCO, ISMPO, JSMO, KSMO, MOS and SSO. *Ann Oncol* 2020; 31: 334-351. 2020/02/19. DOI: 10.1016/j.annonc.2019.12.001.

7. Kudo M, Finn RS, Qin S, et al. Lenvatinib versus sorafenib in first-line treatment of patients with unresectable hepatocellular carcinoma: a randomised phase 3 non-inferiority trial. *Lancet* 2018; 391: 1163-1173. 2018/02/13. DOI: 10.1016/S0140-6736(18)30207-1.

8. Sieghart W, Hucke F and Peck-Radosavljevic M. Transarterial chemoembolization: modalities, indication, and patient selection. *J Hepatol* 2015; 62: 1187-1195. 2015/02/15. DOI: 10.1016/j.jhep.2015.02.010.

9. Lencioni R, de Baere T, Soulen MC, et al. Lipiodol transarterial chemoembolization for hepatocellular carcinoma: A systematic review of efficacy and safety data. *Hepatology* 2016; 64: 106-116. 2016/01/15. DOI: 10.1002/hep.28453.

10. Casadei-Gardini A, Scartozzi M, Tada T, et al. Lenvatinib versus sorafenib in first-line treatment of unresectable hepatocellular carcinoma: An inverse probability of treatment weighting analysis. *Liver Int* 2021; 41: 1389-1397. 2021/02/07. DOI: 10.1111/liv.14817.

11. Fu Z, Li X, Zhong J, et al. Lenvatinib in combination with transarterial chemoembolization for treatment of unresectable hepatocellular carcinoma (uHCC): a retrospective controlled study. *Hepatology international* 2021; 15: 663-675. 2021/04/21. DOI: 10.1007/s12072-021-10184-9.

For peer review only - http://bmjopen.bmj.com/site/about/guidelines.xhtml

12. Kawamura Y, Kobayashi M, Shindoh J, et al. Lenvatinib-Transarterial Chemoembolization Sequential Therapy as an Effective Treatment at Progression during Lenvatinib Therapy for Advanced Hepatocellular Carcinoma. *Liver cancer* 2020; 9: 756-770. 2021/01/15. DOI: 10.1159/000510299.

13. Peng Z, Fan W, Zhu B, et al. Lenvatinib Combined With Transarterial Chemoembolization as First-Line Treatment for Advanced Hepatocellular Carcinoma: A Phase III, Randomized Clinical Trial (LAUNCH). *J Clin Oncol* 2023; 41: 117-127. 2022/08/04. DOI: 10.1200/jco.22.00392.

14. Cai H, Zhang L, Li N, et al. Lenvatinib versus sorafenib for unresectable hepatocellular carcinoma: a cost-effectiveness analysis. *Journal of comparative effectiveness research* 2020; 9: 553-562. 2020/05/19. DOI: 10.2217/cer-2020-0041.

15. Meng R, Zhang X, Zhou T, et al. Cost-effectiveness analysis of donafenib versus lenvatinib for first-line treatment of unresectable or metastatic hepatocellular carcinoma. *Expert Rev Pharmacoecon Outcomes Res* 2022; 22: 1079-1086. 2022/05/18. DOI: 10.1080/14737167.2022.2079498.

16. Zhou T, Wang X, Cao Y, et al. Cost-effectiveness analysis of sintilimab plus bevacizumab biosimilar compared with lenvatinib as the first-line treatment of unresectable or metastatic hepatocellular carcinoma. *BMC health services research* 2022; 22: 1367. 2022/11/19. DOI: 10.1186/s12913-022-08661-4.

17. Chinese Pharmaceutical Association. China Guidelines for Pharmacoeconomic Evaluations. (2019). Available online at: https://www.cpa.org.cn//?do=infoandcid=75017 (accessed May 11, 2022).

18. Guyot P, Ades AE, Ouwens MJ, et al. Enhanced secondary analysis of survival data: reconstructing the data from published Kaplan-Meier survival curves. *BMC Med Res Methodol* 2012; 12: 9. 2012/02/03. DOI: 10.1186/1471-2288-12-9.

19. Meyers BM, Vogel A, Marotta P, et al. The Cost-Effectiveness of Lenvatinib in the Treatment of Advanced or Unresectable Hepatocellular Carcinoma from a Canadian Perspective. *Canadian journal of gastroenterology & hepatology* 2021; 2021: 8811018. 2021/03/09. DOI: 10.1155/2021/8811018.

20. Chen S, Wu Z, Shi F, et al. Lenvatinib plus TACE with or without pembrolizumab for the treatment of initially unresectable hepatocellular carcinoma harbouring PD-L1 expression: a retrospective study. *Journal of cancer research and clinical oncology* 2022; 148: 2115-2125. 2021/08/29. DOI: 10.1007/s00432-021-03767-4.

21. Okusaka T, Ikeda K, Kudo M, et al. Safety and efficacy of lenvatinib by starting dose based on body weight in patients with unresectable hepatocellular carcinoma in REFLECT. *Journal of gastroenterology* 2021; 56: 570-580. 2021/05/06. DOI: 10.1007/s00535-021-01785-0.

22. Li M, Lin S, Wilson L, et al. Cost-Effectiveness Analysis of Hepatic Arterial Infusion of FOLFOX Combined Sorafenib for Advanced Hepatocellular Carcinoma With Portal Vein Invasion. *Frontiers in oncology* 2021; 11: 562135. 2021/03/27. DOI: 10.3389/fonc.2021.562135.

23. Meng R, Cao Y, Zhou T, et al. The Cost Effectiveness of Donafenib Compared With Sorafenib for the First-Line Treatment of Unresectable or Metastatic Hepatocellular

For peer review only - http://bmjopen.bmj.com/site/about/guidelines.xhtml

Carcinoma in China. *Frontiers in public health* 2022; 10: 794131. 2022/04/19. DOI: 10.3389/fpubh.2022.794131.

24. Zhang H, Zeng X, Peng Y, et al. Cost-Effectiveness Analysis of Hepatic Arterial Infusion Chemotherapy of Infusional Fluorouracil, Leucovorin, and Oxaliplatin Versus Transarterial Chemoembolization in Patients With Large Unresectable Hepatocellular Carcinoma. *Front Pharmacol* 2022; 13: 849189. 2022/05/14. DOI: 10.3389/fphar.2022.849189.

25. Zhao M, Pan X, Yin Y, et al. Cost-Effectiveness Analysis of Five Systemic Treatments for Unresectable Hepatocellular Carcinoma in China: An Economic Evaluation Based on Network Meta-Analysis. *Frontiers in public health* 2022; 10: 869960. 2022/05/03. DOI: 10.3389/fpubh.2022.869960.

26. National Health Commission of the People's Republic of China. Guidelines for the diagnosis and treatment of primary hepatic carcinoma (2022 edition). Available online at: http://www.nhc.gov.cn/yzygj/s7659/202201/a01ceb75c62b486fa459e36ba0fdfdbc.shtml (accessed Feb 11, 2023).

27. Wu B, Zhang Q and Sun J. Cost-effectiveness of nivolumab plus ipilimumab as first-line therapy in advanced renal-cell carcinoma. *J Immunother Cancer* 2018; 6: 124. 2018/11/22. DOI: 10.1186/s40425-018-0440-9.

28. Hou Y and Wu B. Atezolizumab plus bevacizumab versus sorafenib as first-line treatment for unresectable hepatocellular carcinoma: a cost-effectiveness analysis. *Cancer communications (London, England)* 2020; 40: 743-745. 2020/11/08. DOI: 10.1002/cac2.12110.

29. NICE Single Technology Appraisal. Lenvatinib for advanced, unresectable, untreated hepatocellular carcinoma: committee papers [TA551].2018. Available online at: https://www.nice.org.uk/guidance/ta551/evidence/appraisal-consultation-committee-paperspdf-6652960238 (Accessed Jun 30, 2021).

For peer review only

For peer review only - http://bmjopen.bmj.com/site/about/guidelines.xhtml

Figure legends:

Figure 1: Tornado diagram of one-way deterministic sensitivity analysis (DSA) of LEN-TACE vs. LEN. The dashed line represents the threshold of willingness-to pay ($37,663/QALYs). ICER, incremental cost-effectiveness ratio; QALYs, quality-adjusted life-year.
Figure 2: Probabilistic sensitivity analysis scatter plot (1,000 iterations). An ellipse surrounds 95% of the estimates. WTP, willingness-to-pay; GDP, gross domestic product.
Figure 3: Cost-effectiveness acceptability curve of LEN-TACE vs. LEN. QALYs, quality-adjusted life-year.

For peer review only

For peer review only - http://bmjopen.bmj.com/site/about/guidelines.xhtml

Table 1: Model parameters.

Item	Base case	Range	Distribution	Source
**Costs (US $)**				
Lenvatinib per 120 mg (PATHEONINC)	499.71	399.77-499.71	Gamma	[25]
Regorafenib per 1,120 mg (Bayer AG)	744.85	372.43- 744.85	Gamma	[25]
Tislelizumab per 100 mg (BeiGene)	223.63	178.91-223.63	Gamma	[25]
TACE per session	1,929	1,543-2,315	Gamma	[24]
Hepatectomy	9,022	7,218-10,827	Gamma	[24]
**Management of adverse events**				
Elevated ALT/AST	56.54	45.23-67.84	Gamma	[23]
Hypertension	35.46	28.36-42.55	Gamma	[23]
Hyperbilirubinemia	113.53	90.82-136.24	Gamma	[15]
Diarrhea	5.66	4.53-6.79	Gamma	[22]
Decreased weight	102.73	98.67- 120.63	Gamma	[15]
**BSC cost per cycle**	265.08	212.06-318.10	Gamma	[25]
**Follow-up and monitoring per month in PFS**	114	86-143	Gamma	[25]
**Follow-up and monitoring per month in PD**	210	157-262	Gamma	[25]
**Terminal care per patient**	1,839	1,519-2,279	Gamma	[25]
**Utility values**				
PFS	0.76	0.61-0.91	Beta	[28]
PD	0.68	0.54-0.82	Beta	[28]
**Disutility of adverse events**				
Elevated ALT/AST	0	NA	NA	[15]
Hypertension	0.012	0.010-0.014	Beta	[15]
Hyperbilirubinemia	0	NA	NA	[15]
Diarrhea	0.047	0.016-0.077	Beta	[22]
Decreased weight	0.053	0.042-0.064	Beta	[15]
**LEN-TACE: Incidence of SAEs**				
Elevated ALT/AST	0.40	0.32-0.48	Beta	[13]
Hypertension	0.20	0.16-0.24	Beta	[13]
Hyperbilirubinemia	0.09	0.072-0.108	Beta	[13]
Diarrhea	0.05	0.04-0.06	Beta	[13]
Decreased weight	0.076	0.06-0.09	Beta	[13]
**LEN: Incidence of SAEs**				
Elevated ALT/AST	0.03	0.024-0.036	Beta	[13]
Hypertension	0.20	0.16-0.24	Beta	[13]
Hyperbilirubinemia	0.03	0.024-0.036	Beta	[13]
Diarrhea	0.04	0.032-0.048	Beta	[13]
Decreased weight	0.07	0.056-0.084	Beta	[13]
**Discount rate(%)**	5	0-8	Fixed in PSA	
**Proportion of receiving subsequent treatments in LEN-TACE**				
Hepatectomy	0.16	0.128-0.192	Beta	[13]
Regorafenib	0.22	0.176-0.264	Beta	[13[
Tislelizumab	0.43	0.344-0.516	Beta	[13]
BSC	0.19	0.152-0.228	Beta	[13]
**Proportion of receiving subsequent treatments in LEN**				
TACE	0.07	0.056-0.084	Beta	[13]
Regorafenib	0.25	0.20-0.30	Beta	[13]
Tislelizumab	0.51	0.408-0.612	Beta	[13]
BSC	0.17	0.136-0.204	Beta	[13]
**LEN treatment duration (months)**	15.0	8.0-15.0	Normal	[13]

BSC, best supportive care; PFS, progression-free survival; PD, progressed disease; PSA, probabilistic sensitivity analysis; LEN, lenvatinib; TACE, transarterial chemoembolization; SAEs, serious adverse events.

For peer review only - http://bmjopen.bmj.com/site/about/guidelines.xhtml
For peer review only

Table 2: Base-case and scenario analyses results.

	Treatment	Cost ($)	Δ cost	QALYs	Δ QALYs	ICER
**Base-case**						
	LEN-TACE	31,394	12,151	1.166	0.382	31,808
	LEN	19,243	NA	0.784	NA	NA
**Scenario**						
Weibull model	LEN-TACE	30,629	11,736	1.072	0.328	35,780
	LEN	18,893	NA	0.744	NA	NA
Royston/Parmar spline model	LEN-TACE	31,468	10,733	1.203	0.320	33,540
	LEN	20,735	NA	0.883	NA	NA

QALYs, quality-adjusted life years; ICER, incremental cost-effectiveness ratio; NA, not applicable; LEN, lenvatinib; TACE, transarterial chemoembolization.

For peer review only

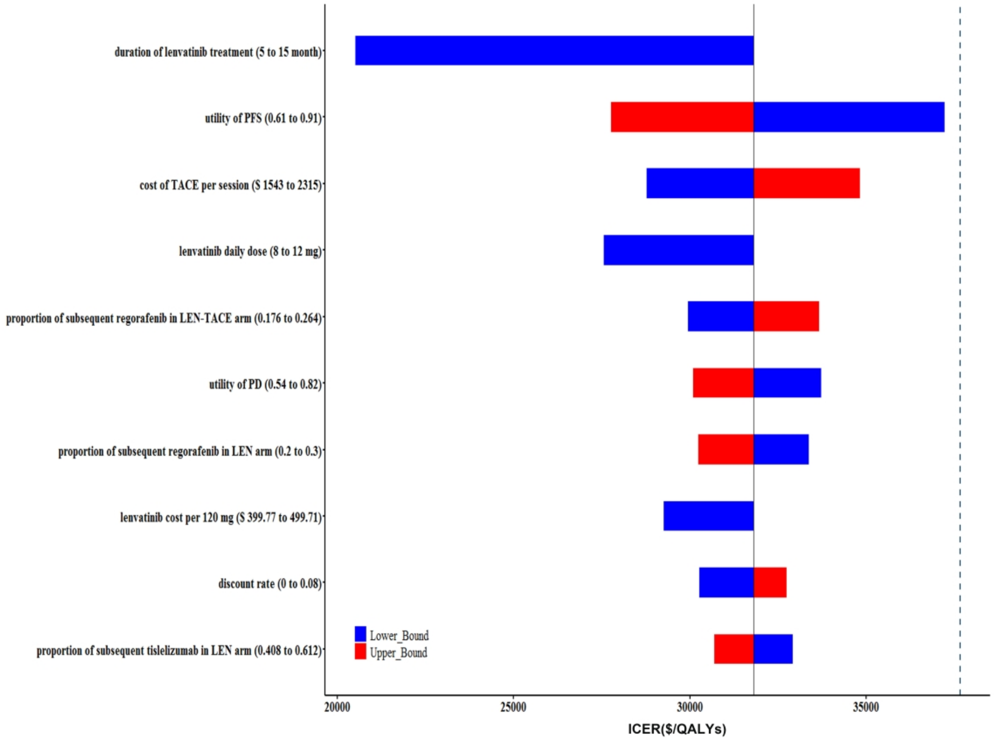

Figure 1: Tornado diagram of one-way deterministic sensitivity analysis (DSA) of LEN-TACE vs. LEN. The dashed line represents the threshold of willingness-to pay ($37,663/QALYs). ICER, incremental cost-effectiveness ratio; QALYs, quality-adjusted life-year.

129x96mm (1200 x 1200 DPI)

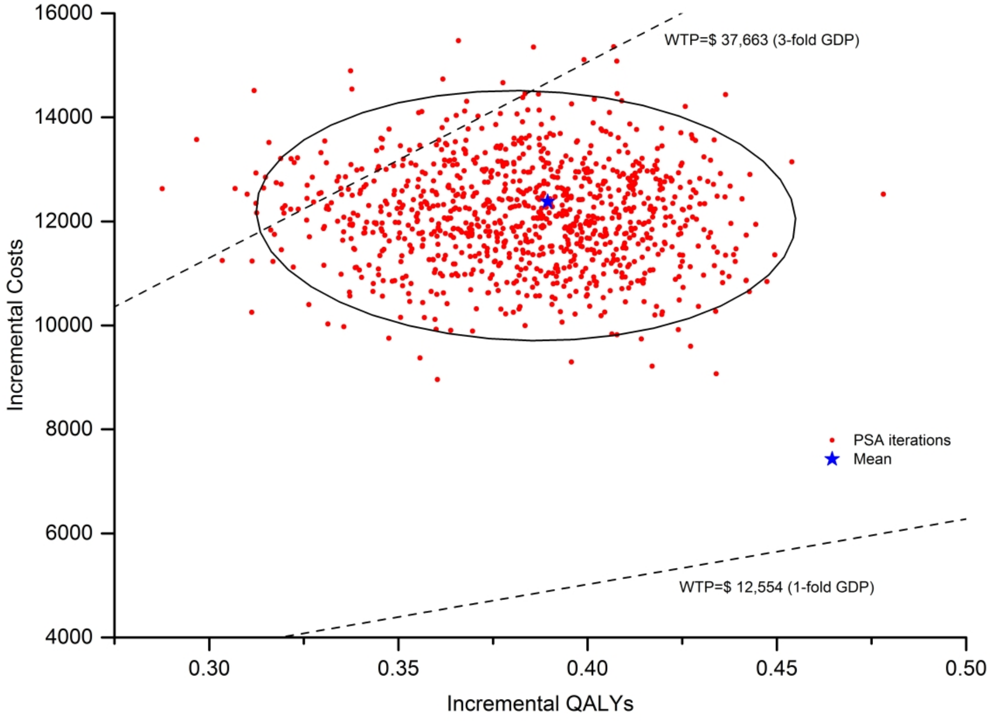

Figure 2: Probabilistic sensitivity analysis scatter plot (1,000 iterations). An ellipse surrounds 95% of the estimates. WTP, willingness-to-pay; GDP, gross domestic product.

231x168mm (600 x 600 DPI)

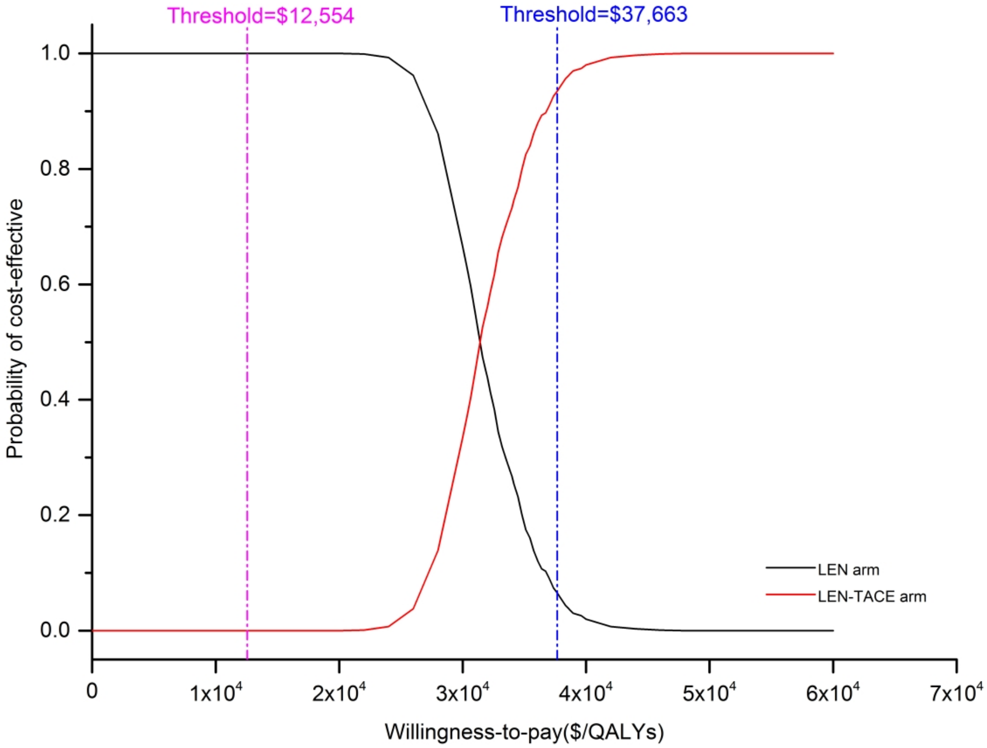

Figure 3: Cost-effectiveness acceptability curve of LEN-TACE vs. LEN. QALYs, quality-adjusted life-year.

227x174mm (600 x 600 DPI)

Supplementary Table S1: Fit parameters, AIC and BIC values for each model

			LEN-TACE				LEN			
**Index**	**Distribution**	**Parameter**	**Est**	**Se**	**AIC**	**BIC**	**Est**	**Se**	**AIC**	**BIC**
**PFS**	Log-logistic	shape	3.2270	0.2480	758.94*	765.21*	2.9320	0.2030	815.61*	821.86*
		scale	10.8880	0.4870			6.6220	0.3100		
	Gompertz	shape	0.1143	0.0152	806.42	812.70	0.0772	0.0145	879.20	885.45
		rate	0.0271	0.0048			0.0762	0.0099		
	Weibull	shape	2.1600	0.1540	775.03	781.30	1.7390	0.1050	841.79	848.04
		scale	13.7820	0.5960			8.8380	0.4360		
	Log-normal	meanlog	2.3910	0.0470	762.04	768.31	1.8878	0.0486	817.74	823.99
		sdlog	0.5513	0.0371			0.6067	0.0360		
	Gamma	shape	3.9865	0.4942	764.58	770.86	3.0411	0.3359	826.29	832.54
		rate	0.3246	0.0457			0.3888	0.0481		
	Exponential	rate	0.0671	0.0063	853.26	856.40	0.1198	0.0100	901.13	904.25
	Gen-gamma	mu	2.4161	0.0739	763.85	773.26	1.8818	0.0776	819.73	829.10
		sigma	0.5424	0.0421			0.6073	0.0365		
		Q	0.1055	0.2432			-0.0207	0.2082		
	Royston/Parmar spline model(scale=normal, 0 kont)	gamma0	-4.3370	0.2890	762.04	768.31	-3.1117	0.1961	817.74	823.99
		gamma1	1.8140	0.1220			1.6483	0.0978		
	Royston/Parmar spline model(scale=normal, 1 kont)	gamma0	-4.0295	0.5378	763.60	773.00	-3.0669	0.3074	819.71	829.08
		gamma1	1.6159	0.3179			1.6082	0.2342		
		gamma2	-0.0896	0.1345			-0.0128	0.0682		
	Royston/Parmar spline model(scale=normal, 2 kont)	gamma0	-3.4563	0.5592	762.03	774.57	-2.7900	0.3280	818.99	831.49
		gamma1	1.1548	0.3642			1.2590	0.2970		
		gamma2	-1.7142	0.8676			-0.7910	0.4710		
		gamma3	2.0204	1.0765			0.8290	0.5030		
	Royston/Parmar spline model(scale=normal, 3 kont)	gamma0	-3.3430	0.5660	763.68	779.36	-2.8390	0.3470	820.77	836.39
		gamma1	1.0410	0.3910			1.3370	0.3380		
		gamma2	-1.9230	1.9900			0.1340	1.4000		
		gamma3	1.7270	4.0660			-1.3300	2.5880		
		gamma4	0.2800	2.7960			1.3640	1.4860		
	Royston/Parmar spline model(scale=normal, 4 kont)	gamma0	-3.3579	0.5768	765.68	765.68	-2.9480	0.3750	821.45	840.20
		gamma1	1.0583	0.4067			1.5200	0.3900		
		gamma2	-1.3814	3.7579			1.9930	1.8950		
		gamma3	-0.3330	11.2152			-6.3750	5.798		
		gamma4	1.7765	11.3547			4.8450	5.7250		
		gamma5	0.0639	4.6610			-0.2470	1.9700		
	Royston/Parmar spline model(scale=normal, 5 kont)	gamma0	-3.3840	0.5890	766.75	766.75	-3.1760	0.4280	820.02	841.89
		gamma1	1.0830	0.4220			1.8950	0.4830		
		gamma2	1.3230	5.3950			4.0920	1.9290		
		gamma3	-12.2830	19.0650			-12.5980	6.4900		
		gamma4	17.4970	22.9360			15.0020	10.1850		
		gamma5	-10.8000	15.3220			-8.4620	7.7910		
		gamma6	5.0130	7.5430			2.6350	2.7250		
**OS**	Log-logistic	shape	3.3020	0.3160	582.22*	588.49*	2.9400	0.2380	723.78*	730.03*
		scale	17.9700	0.9050			11.7940	0.5960		
	Gompertz	shape	0.1391	0.0180	598.10	604.37	0.0811	0.0145	773.36	779.61
		rate	0.0073	0.0020			0.0298	0.0052		
	Weibull	shape	2.6100	0.2420	584.79	591.06	1.9440	0.1450	743.98	750.22
		scale	21.1940	0.9790			15.4080	0.7770		

For peer review only

Supplementary Table S1: Fit parameters, AIC and BIC values for each model

			LEN-TACE				LEN			
**Index**	**Distribution**	**Parameter**	**Est**	**Se**	**AIC**	**BIC**	**Est**	**Se**	**AIC**	**BIC**
	Log-normal	meanlog	2.8937	0.0569	585.65	591.92	2.4786	0.0538	726.80	733.05
		sdlog	0.5573	0.0470			0.6080	0.0431		
	Gamma	shape	4.4499	0.6680	583.19	589.47	3.3508	0.4286	732.93	739.18
		rate	0.2273	0.0408			0.2445	0.0367		
	Exponential	rate	0.0331	0.0038	654.31	657.45	0.0589	0.0058	799.13	802.25
	Gen-gamma	mu	2.9861	0.0734	585.14	594.55	2.4493	0.0893	728.63	738.00
		sigma	0.4595	0.0726			0.6167	0.0481		
		Q	0.5504	0.3377			-0.1120	0.2702		
	Royston/Parmar spline model(scale=normal, 0 kont)	gamma0	-5.1930	0.4100	585.65	591.92	-4.0770	0.2830	726.80	733.05
		gamma1	1.7950	0.1510			1.6450	0.1170		
	Royston/Parmar spline model(scale=normal, 1 kont)	gamma0	-3.9839	0.6927	583.99	593.40	-4.0452	0.5118	728.79	738.16
		gamma1	1.1578	0.3387			1.6253	0.2871		
		gamma2	-0.4984	0.2530			-0.0106	0.1433		
	Royston/Parmar spline model(scale=normal, 2 kont)	gamma0	-3.9100	0.7600	586.01	598.55	-3.3200	0.5010	723.12	735.61
		gamma1	1.1080	0.4000			1.0510	0.3100		
		gamma2	-0.5850	1.6860			-2.4310	0.8620		
		gamma3	0.2710	2.6460			3.0190	1.0760		
	Royston/Parmar spline model(scale=normal, 3 kont)	gamma0	-4.4770	0.9470	585.36	601.04	-3.5370	0.5570	721.47	737.09
		gamma1	1.4830	0.5240			1.2520	0.3600		
		gamma2	3.2720	2.2140			2.3380	2.5060		
		gamma3	-13.3810	8.0390			-8.6030	5.0560		
		gamma4	13.1590	8.3970			8.1020	3.3600		
	Royston/Parmar spline model(scale=normal, 4 kont)	gamma0	-4.4870	0.9800	587.42	587.42	-3.7100	0.6260	721.69	740.43
		gamma1	1.4910	0.5490			1.4040	0.4300		
		gamma2	2.6710	2.5600			1.6380	2.4290		
		gamma3	-5.4500	15.3950			-2.2890	8.3800		
		gamma4	-4.4210	25.1920			-6.9260	11.6520		
		gamma5	11.0260	16.5950			10.0550	6.3980		
	Royston/Parmar spline model(scale=normal, 5 kont)	gamma0	-4.3490	0.9860	589.24	589.24	-3.9170	0.6910	719.66	741.52
		gamma1	1.3990	0.5610			1.5940	0.4900		
		gamma2	0.2810	2.9780			4.2290	2.8250		
		gamma3	9.2650	16.6470			-15.6820	11.1400		
		gamma4	-23.8210	38.8820			25.5560	19.8710		
		gamma5	9.1510	45.3570			-32.2010	20.6110		
		gamma6	10.8110	27.9880			21.7010	10.4480		

*, best fitted model; PFS, progression-free survival; OS, overall survival; AIC, Akaike information criterion; BIC, Bayesian information criterion.

For peer review only

Supplementary Table S2: Validation of modeled PFS and OS data using internal and external data.

Curve	6-month (%)	12-month (%)	24-month (%)	36-month (%)
**PFS**				
LEN-TACE				
log-logistic	87.2	42.2	7.2	2.1
weibull	84.7	47.6	3.6	0.035
royston/parmar spline model (knot=5)	88.5	41.7	9.5	3.2
LAUNCH	88.2	39.2	11.4	NA
LEN				
log-logistic	57.2	14.9	2.2	0.7
weibull	60.0	18.2	0.3	0.001
royston/parmar spline model (knot=5)	56.9	14.2	2.9	1.0
LAUNCH	54.8	14.3	2.5	NA
**OS**				
LEN-TACE				
log-logistic	97.4	79.1	27.8	9.2
weibull	96.4	79.7	25.1	1.9
royston/parmar spline model (knot=5)	96.7	80.9	28.3	12.1
Chen Song et al, 2022	84.9	63.5	18.5	9.5
LAUNCH	95.9	81.5	26.1	NA
LEN				
log-logistic	87.9	48.7	11.0	3.6
weibull	85.2	54.1	9.4	0.6
royston/parmar spline model (knot=2)	89.8	45.0	16.2	8.0
REFLECT	82.1	55.0	29.8	13.0
LAUNCH	87.7	47.0	17.8	NA

PFS, progression-free survival; OS, overall survival; NA, not applicable.

For peer review only

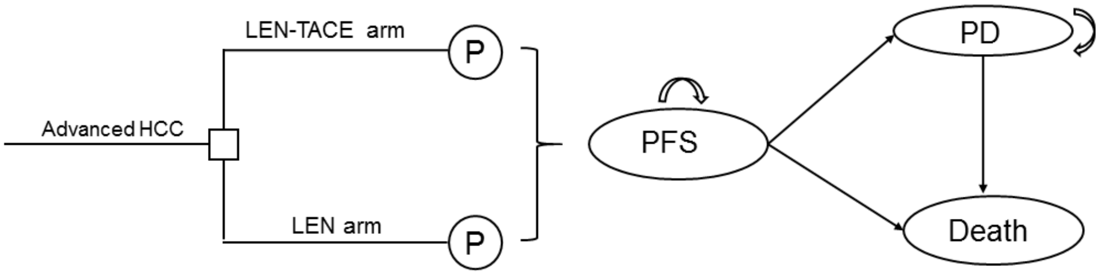

Supplementary Figure S1: Model structure of a decision tree combining the partitioned survival model. (HCC, hepatocellular carcinoma; LEN, lenvatinib; TACE, transarterial chemoembolization; PFS, progression-free survival; PD, progressed survival).

For peer review only

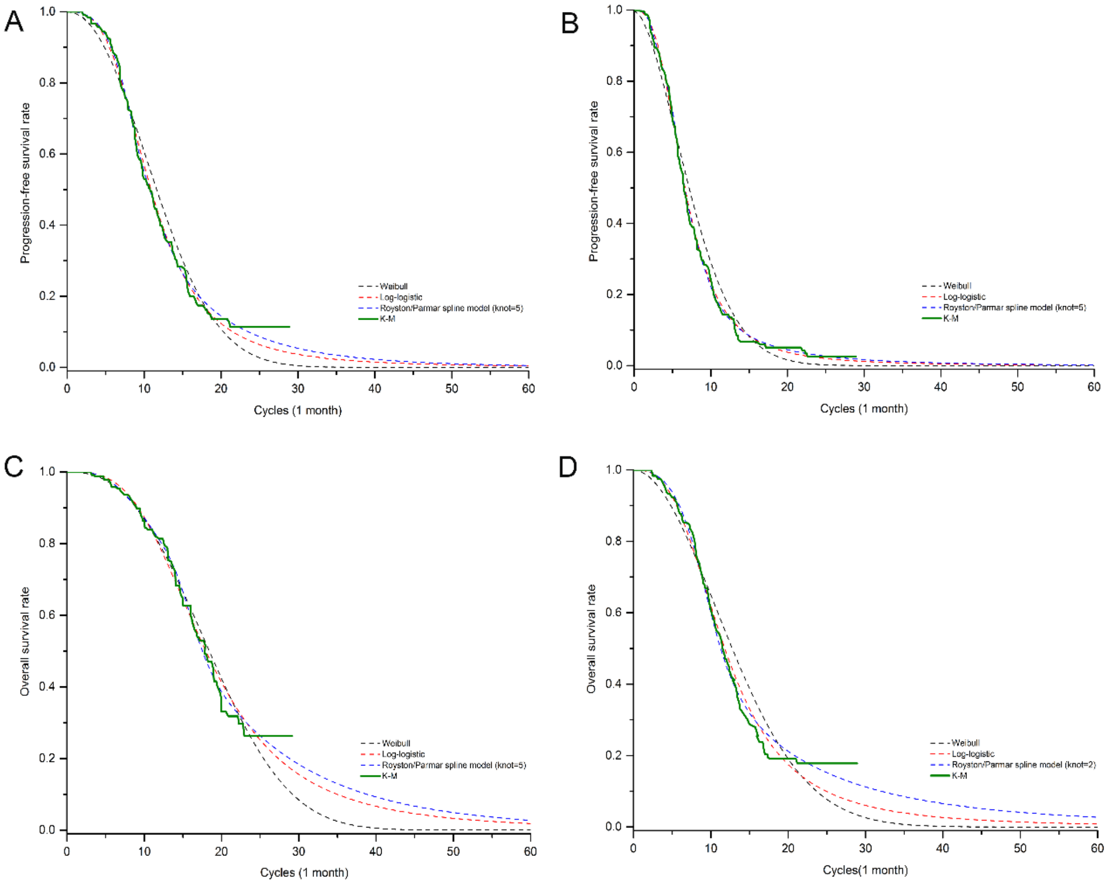

Supplementary Figure S2: The exploration and fitting of PFS and OS curves in the partitioned survival model. (A) PFS of LEN-TACE arm, (B) PFS of LEN arm, (C) OS of LEN-TACE arm, (D) OS of LEN arm.

For peer review only - http://bmjopen.bmj.com/site/about/guidelines.xhtml

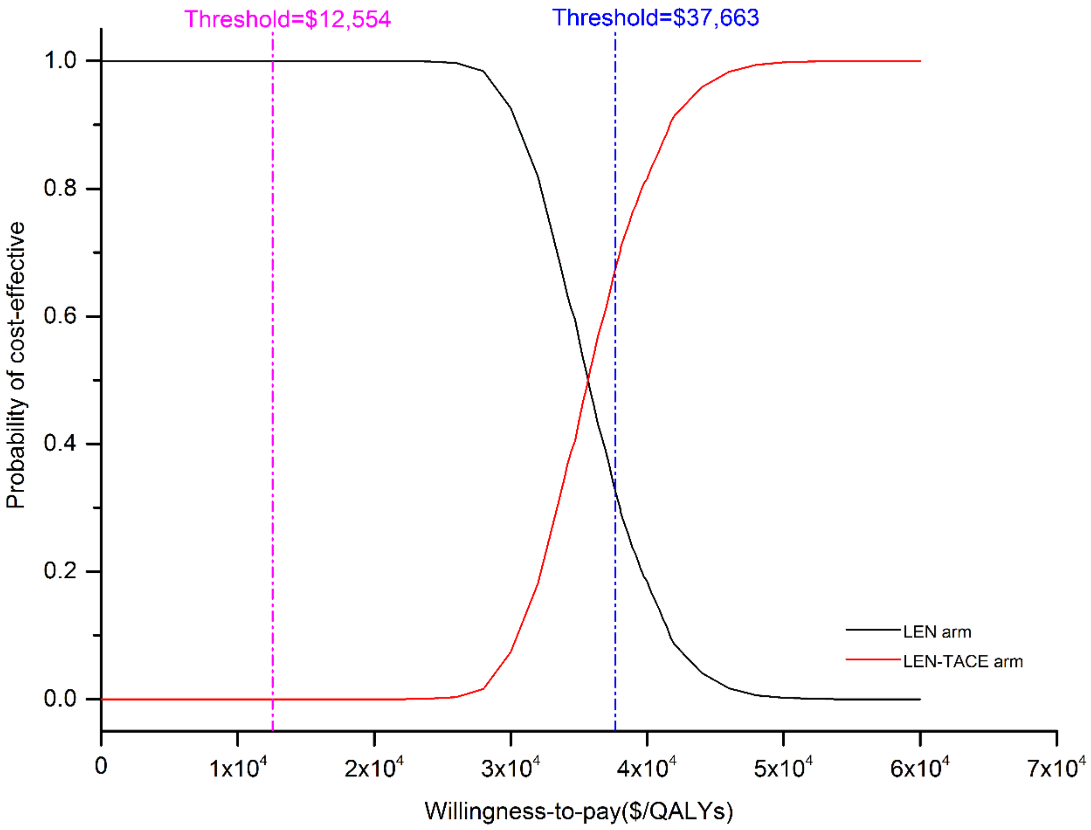

Supplementary Figure S3: Cost-effectiveness acceptability curve of LEN-TACE vs. LEN adopting Weibull distribution model. QALYs, quality-adjusted life-year.

review only

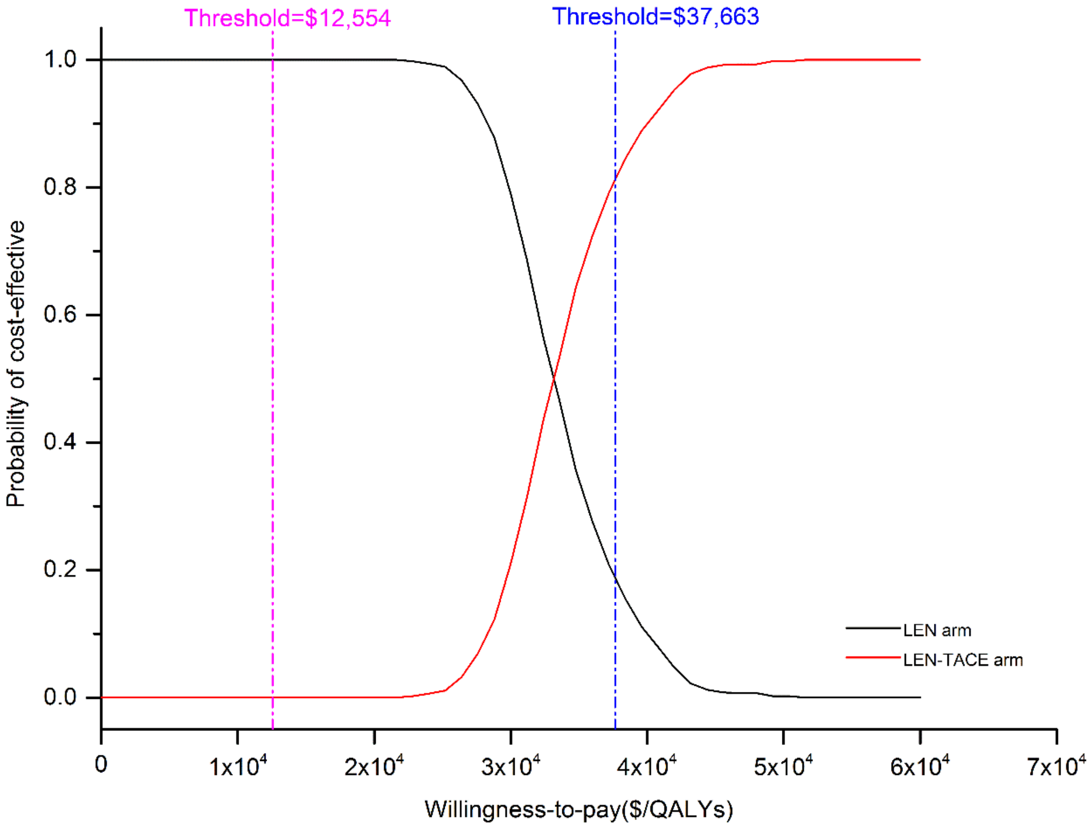

Supplementary Figure S4: Cost-effectiveness acceptability curve of LEN-TACE vs. LEN adopting Royston/Parmar spline model. QALYs, quality-adjusted life-year.

review only

CHEERS 2022 checklist			
Section/topic	Item no.	Guidance for reporting	Reported in section
Title	1	Identify the study as an economic evaluation and specify the interventions being compared.	Page 2
Abstract			
Abstract	2	Provide a structured summary that highlights context, key methods, results, and alternative analyses.	Page 3
Introduction			
Background and objectives	3	Give the context for the study, the study question, and its practical relevance for decision making in policy or practice.	Page 4
Methods			
Health economic analysis plan	4	Indicate whether a health economic analysis plan was developed and where available.	Page 4-5
Study population	5	Describe characteristics of the study population (such as age range, demographics, socioeconomic, or clinical characteristics).	Page 5
Setting and location	6	Provide relevant contextual information that may influence findings.	Page 5
Comparators	7	Describe the interventions or strategies being compared and why chosen.	Page 5
Perspective	8	State the perspective(s) adopted by the study and why chosen.	Page 5
Time horizon	9	State the time horizon for the study and why appropriate.	Page 4
Discount rate	10	Report the discount rate(s) and reason chosen.	Page 5
Selection of outcomes	11	Describe what outcomes were used as the measure(s) of benefit(s) and harm(s).	Page 5
Measurement of outcomes	12	Describe how outcomes used to capture benefit(s) and harm(s) were measured.	Page 5
Valuation of outcomes	13	Describe the population and methods used to measure and value outcomes.	Page 5-6
Measurement and valuation of resources and costs	14	Describe how costs were valued.	Page 5-6
Currency, price date, and conversion	15	Report the dates of the estimated resource quantities and unit costs, plus the currency and year of conversion.	Page 5
Rationale and description of model	16	If modeling is used, describe in detail and why used. Report if the model is publicly available and where it can be accessed.	Page 5
Analytics and assumptions	17	Describe any methods for analysing or statistically transforming data, any extrapolation methods, and	Page 5 ,6, 8

For peer review only

		approaches for validating any model used.	
Characterizing heterogeneity	18	Describe any methods used for estimating how the results of the study vary for subgroups.	Page 6-7
Characterizing distributional effects	19	Describe how impacts are distributed across different individuals or adjustments made to reflect priority populations.	Page 6-7
Characterizing uncertainty	20	Describe methods to characterize any sources of uncertainty in the analysis.	Page 6-7
Approach to engagement with patients and others affected by the study	21	Describe any approaches to engage patients or service recipients, the general public, communities, or stakeholders (such as clinicians or payers) in the design of the study.	Page 7
Results			
Study parameters	22	Report all analytic inputs (such as values, ranges, references) including uncertainty or distributional assumptions.	Page 14, 19
Summary of main results	23	Report the mean values for the main categories of costs and outcomes of interest and summarize them in the most appropriate overall measure.	Page 15
Effect of uncertainty	24	Describe how uncertainty about analytic judgments, inputs, or projections affect findings. Report the effect of choice of discount rate and time horizon, if applicable.	Page16-18, 21, 23-25
Effect of engagement with patients and others affected by the study	25	Report on any difference patient/service recipient, general public, community, or stakeholder involvement made to the approach or findings of the study	Page 6
Discussion			
Study findings, limitations, generalizability, and current knowledge	26	Report key findings, limitations, ethical or equity considerations not captured, and how these could affect patients, policy, or practice.	Page 7-8
Other relevant information			
Source of funding	27	Describe how the study was funded and any role of the funder in the identification, design, conduct, and reporting of the analysis	Page 8
Conflicts of interest	28	Report authors conflicts of interest according to journal or International Committee of Medical Journal Editors requirements.	Page 8
From: Husereau D, Drummond M, Augustovski F, et al. Consolidated Health Economic Evaluation Reporting Standards 2022 (CHEERS 2022) Explanation and Elaboration: A Report of the ISPOR CHEERS II Good Practices Task Force. Value Health 2022;25. doi:10.1016/j.jval.2021.10.008			

For peer review only

For peer review only
